# Winter GPS tagging reveals home ranges during the breeding season for a boreal-nesting migrant songbird, the Golden-crowned Sparrow

**DOI:** 10.1371/journal.pone.0305369

**Published:** 2024-06-12

**Authors:** Autumn R. Iverson, Diana L. Humple, Renée L. Cormier, Thomas P. Hahn, Theadora A. Block, Daizaburo Shizuka, Bruce E. Lyon, Alexis S. Chaine, Emily J. Hudson, Elisha M. Hull

**Affiliations:** 1 Department of Animal Science, University of California Davis, Davis, California, United States of America; 2 Point Blue Conservation Science, Petaluma, California, United States of America; 3 Department of Neurobiology, Physiology and Behavior, University of California Davis, Davis California, United States of America; 4 Department of Ecology and Evolutionary Biology, University of California, Santa Cruz, California, United States of America; 5 School of Biological Sciences, University of Nebraska-Lincoln, Lincoln, Nebraska, United States of America; 6 Station d’Ecologie Théorique et Expérimentale du CNRS (UAR2029), Evolutionary Ecology Group, Moulis, France; 7 Institute for Advanced Studies in Toulouse, Toulouse School of Economics, Toulouse, France; Bowling Green State University, UNITED STATES

## Abstract

Determining space use for species is fundamental to understanding their ecology, and tracking animals can reveal insights into their spatial ecology on home ranges and territories. Recent technological advances have led to GPS-tracking devices light enough for birds as small as ~30 g, creating novel opportunities to remotely monitor fine-scale movements and space use for these smaller species. We tested whether miniaturized GPS tags can allow us to understand space use of migratory birds away from their capture sites and sought to understand both pre-breeding space use as well as territory and habitat use on the breeding grounds. We used GPS tags to characterize home ranges on the breeding grounds for a migratory songbird with limited available breeding information, the Golden-crowned Sparrow (*Zonotrichia atricapilla*). Using GPS points from 23 individuals across 26 tags (three birds tagged twice), we found home ranges in Alaska and British Columbia were on average 44.1 ha (95% kernel density estimate). In addition, estimates of territory sizes based on field observations (mean 2.1 ha, 95% minimum convex polygon [MCP]) were three times smaller than 95% MCPs created using GPS tags (mean 6.5 ha). Home ranges included a variety of land cover classes, with shrubland particularly dominant (64–100% of home range cover for all but one bird). Three birds tracked twice returned to the same breeding area each year, supporting high breeding site fidelity for this species. We found reverse spring migration for five birds that flew up to 154 km past breeding destinations before returning. GPS-tracking technology allowed for critical ecological insights into this migratory species that breeds in very remote locations.

## Introduction

Animal space use and movement is important to understand for a variety of ecological and evolutionary processes [1] and in recent years, these studies are increasingly contributing to conservation management [2]. Animal space use is often defined through home ranges and territories [3, 4]. Home ranges are classically defined as the area an individual uses for its “normal activities,” such as foraging, mating, and raising young [5], but this definition is broad and has limited utility for quantifying home range sizes [6, 7]. Territories are considered areas within the home range that are defended from competitors [5, 8] but defining territories has not been consistent across studies [3]. Despite the lack of consensus, the characterization of different types of spatial behavior based on these functional definitions of home range and territory have been an important theme of inquiry in movement ecology [9]. Quantifying the relationship between home range and territory size is important in the context of ecology and conservation and can lead to a better understanding of population dynamics, resource use, and species demographics.

In the field, it can be difficult to gather information on the full home range and therefore to distinguish accurately between a home range and territory. For passerines on the breeding grounds, a common research approach is to delineate territories, often through mapping the movements of singing males [10, 11]. In temperate regions, male passerines often sing to establish a breeding territory which they use to attract mates, ensure paternity, and defend food resources [12]. However, during the breeding season, both males and females leave territories for several reasons, including extra-pair copulations and/or for additional foraging opportunities [13, 14]. This larger space-use could be considered the home range [5] but delineating this area can be difficult because extra-territorial movements may last for short periods and involve secretive behavior. Previous studies suggest that home ranges are generally much larger than territories. For example, radio telemetry has shown that birds often use resources outside of territories, including Cerulean Warblers (*Setophaga cerulea*) with home ranges significantly larger than territories [15], and Golden-winged Warblers (*Vermivora chrysoptera*) with home ranges 2–4 times as large as spot-mapped territories and 40% of telemetry locations outside these territories [16]. Similarly, territories of male Nightingales (*Luscinia megarhynchos*) made up only 50% of their total activity area, although they spent 90% of their time within the territory [17].

Even if birds spend less time outside their territory than within, their larger home range is important for their breeding life history and can also be important in a conservation context. For example, male Hooded Warblers (*Wilsonia citrina*) traveled outside their territories for extra-pair copulations but were limited in opportunities by forest fragmentation [14]. Some benefits of extra-pair copulations can include increasing male reproductive success, potentially providing fertility insurance for females, and improving genetic diversity (reviewed in [18]). Space beyond the territory may also be important for obtaining resources, as in male Chaffinches seen foraging when outside singing territories [19]. Large movements during the breeding season may also serve information gathering purposes for future dispersal, as shown for Kirtland’s warblers (*Setophaga kirtlandii* [20]). With many passerine populations declining [21, 22], conservation efforts on the breeding grounds will be most effective when focused on complete home ranges, as these areas may be serving important functions for individual and population survival and reproductive fitness. Further, by understanding habitat use across different land cover types and the connectivity among those habitat types while on the breeding grounds, conservation activities can more effectively support the broad habitat needs of species.

Recent technological advances have led to GPS-tracking devices light enough to be outfitted to some small birds (reviewed in [23]), providing new opportunities to study breeding-season movements outside territories and to describe a full home range area [24, 25]. One major advantage of GPS tracking is that migratory birds tagged during one season can be passively tracked during other times of the year, including in remote areas that are difficult to access, enabling a large array of new ecological research on species and life-stages that are difficult to observe or access. GPS tracking therefore offers an opportunity to study boreal birds breeding in remote areas, many of which are at a higher risk of negative impacts from climate change due to substantial reductions in suitable habitat expected over the coming decades [26].

Golden-crowned Sparrows (*Zonotrichia atricapilla*) breed in parts of Alaska, the Yukon, British Columbia, and Alberta. They breed in forest-tundra ecotones and shrubby-tundra habitats, but there is limited information on many aspects of the natural history of this species at the breeding grounds [27]. For example, information on pair formation, nest construction, territory size, or quantitative analyses of nest site characteristics is missing or understudied [27], and to our knowledge no previous estimates of home range sizes on the breeding grounds have been reported. Similarly, fundamental information such as the space and habitat use of adults on their breeding home ranges is poorly understood [27]. Here, we test whether miniaturized GPS tags deployed on birds on their wintering grounds can allow us to characterize remote home ranges for Golden-crowned Sparrows in/near the boreal forests of Alaska and Canada. To investigate space use, we compare home range estimates obtained from GPS tags to home range estimates from spot-mapping in the field. We expected that GPS tags would allow us to discern movements and spatial use on home ranges larger in area than estimates based on field observations alone. We also expected new insights into home range characterization from GPS tags and we investigated differences in home range sizes based on sex, elevation, latitude, and habitat type.

## Materials and methods

### Study sites and capture

**Fig 1:**
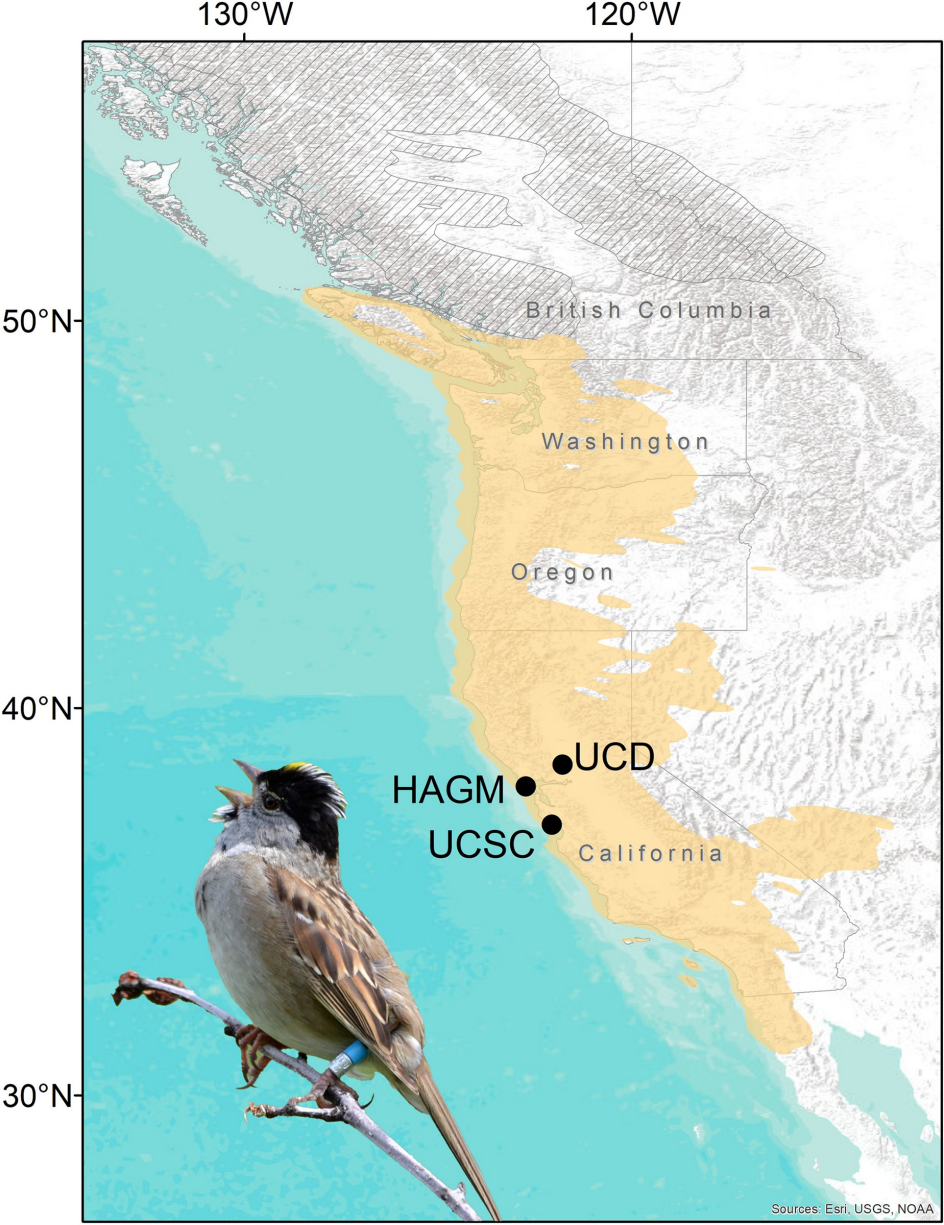
Study sites (black dots) for Golden-crowned Sparrows (*Zonotrichia atricapilla*, inset) on the wintering grounds (beige color) during winters 2017–2021. The southern extent of the breeding range is partially shown with gray hatching. The range map image was provided by eBird (www.ebird.org) and created 9 May 2024. UCD = University of California, Davis; HAGM = Hagmaier Ranch, Olema Valley, Point Reyes National Seashore; UCSC = University of California, Santa Cruz. Inset photo credit: Theadora Block.

Animal capture and handling was reviewed and approved by the University of California Davis Institutional Animal Care and Use Committee Protocol (IACUC) 20823, the University of California Santa Cruz IACUC 1808, the University of Nebraska Lincoln IACUC 1626, and the Palomarin Field Station Animal Care and Use Protocol (vers. 1.1; United States Federal Bird Banding permits 09316 to Diana Humple, 22712 to Thomas Hahn, 10516 to Bruce Lyon, and 23579 to Dai Shizuka). Birds were released within an hour at the site of capture or sooner if displaying signs of stress, and no birds died while being handled for this study. We captured birds at three study sites within the wintering range from 2017–2021 (Fig 1) in western regions of the USA, where GPS tags were deployed and retrieved the following winter. Specifically, birds were caught at three California sites: (1) from 19 Feb 2019 to 30 Jan 2021 at the Zoology Field Building on the University of California, Davis campus, Yolo County (UCD, 38.528, -121.782), (2) from 3 Jan 2019 to 9 Jan 2021 at Hagmaier Ranch in Olema Valley, Point Reyes National Seashore, Marin County, in the San Francisco Bay Area (HAGM, 37.971, -122.731), and (3) from 1 Oct 2017 to 1 Nov 2018 at the University of California Santa Cruz Arboretum, Santa Cruz County (UCSC, 36.983, -122.060). At each site we used Potter traps baited with seed (millet or mixed seeds) and/or passive mist nets to capture birds. For each bird captured, we banded them (with a federal band and color bands) and recorded mass, age, fat score (on a scale from 0–7 for HAGM and UCD and 0–3 at UCSC), and wing chord length. We determined age by plumage [28], particularly the crown plumage (following [29]), or by timing of original capture for recaptured birds (UCSC site only).

### Genetic sexing

We collected blood for genetic sexing at UCSC; at UCD and HAGM we collected feathers for genetic sexing, and for stable isotope analysis of diet as part of a related migratory connectivity study. For individuals at each feather collection site, we collected 8–10 contour feathers for genetic sexing and two outer tail feathers (rectrices) for stable isotope analysis. We sent contour feathers to Animal Genetics, Inc (Tallahassee, FL). Blood samples (*n* = 4) were processed by the Shizuka lab at the School of Biological Sciences at the University of Nebraska-Lincoln, and birds sexed by amplifying the CHD gene on the W and Z chromosomes following [30, 31]. All birds with retrieved GPS tags were sexed (*n* = 23; three birds GPS-tagged twice). Feather and blood sampling for GPS-tagged birds happened upon tag deployment at the UCSC field site, and during retrieval for the other two sites.

### GPS tag deployment and data filtering

We deployed 120 archival 1.0–1.2 g GPS tags with an expected horizontal accuracy within 10 m (PinPoint-10 GPS Store on Board tags from Lotek Wireless; tag mass varied slightly after manufacturing). We deployed 33 tags at UCD, 17 at HAGM, and 70 at UCSC (Fig 1). For seven of 10 returned individuals at UCD in 2019, we gave them a replacement tag for another year of tracking. For all tags, a known location was collected before deployment, which was used for post-processing “Swift” fixes (see S1 File), an algorithm developed by Lotek Wireless to get more efficient GPS fixes and that delivers a greater number of fixes for a given amount of (limited) battery life [32].

Tags were attached using leg-loop harnesses [33] with Stretch Magic jewelry cord as reported previously [34]. To determine eligibility for a tag at the UCSC site, we only put tags on birds weighing more than 25 grams (<4% of body mass). For the UCD and HAGM sites, to determine eligibility for a tag, we 1) determined tag mass, and 2) calculated a lean body mass for each bird by subtracting a coarse estimated mass of fat, as adapted from lean mass guidelines [28] for White-crowned Sparrows (*Zonotrichia leucophrys*) modified for use by Point Blue Conservation Science (see [34] for details). The combined tag and harness mass needed to be less than 5% of the bird’s lean body mass (range = 3.2–4.6%, mean ± SD = 3.8% ± SD 0.4%).

At UCD and HAGM, tags were programmed to collect GPS fixes (hereafter “points”) every ninth day during the estimated breeding season (Jun 2-Aug 31) in 2019 and every thirteenth day during a refined estimated breeding season (May 29-Aug 28; refined after assessing the 2019 data) in 2020. Each tag attempted to retrieve points in the morning, between 16:00 and 16:30 UTC (8:00–9:30 am local time). We set tags to attempt points for up to 12 seconds and then if not successful (e.g., if the bird was under dense vegetation), attempts stopped for the day to conserve battery life. For the UCSC birds, tags were programmed to take points every two to five days (see [35] for programming details). In all cases, we programmed tags to collect as many points as possible while considering the life of the batteries and other tracking needs (e.g., migration), as increasing the number of points can lead to better home range estimates.

We classified GPS points as “breeding” points (i.e., on the breeding grounds regardless of actual breeding status which we could not confirm) to use for home range analyses (described below) if they were within a cluster of points between spring and fall migration. This switch between spring migration and breeding points was determined visually, as migration points showed directed movement averaging 100–120 km/day [34] and at the breeding grounds birds stayed within a much more restricted area (points within ~1–2 km) until fall migration was initiated.

When GPS data were downloaded after tag retrieval, each estimated point was reported with measures of accuracy that, when combined, informed the probability that a point had high or low accuracy. One measure of accuracy is the Horizontal Dilution of Precision (HDOP) which is based on the location and geometry of the satellites used to calculate the tag position; HDOP values of >20 are considered poor, 10–20 are defined as fair, 5–10 are considered moderate, and anything up to five is good to ideal [32]. The data on each tag also included the number of satellites used to obtain the point, with more satellites providing higher location accuracy; specifically, five or more satellites provide the highest accuracy while the minimum number of satellites (three satellites) gives only a 2D fix (no altitude measure [32]). However, both HDOP and the number of satellites only measure the probability of accuracy and are not a guarantee of accuracy, and points with high accuracy can sometimes have higher HDOP values (Lotek Wireless pers. comm.). Overall, Lotek reports an average accuracy for these tags at ± 10 m. Based on these probabilities of accuracy, and to ensure retaining the most points with a high probability of accuracy for home range analyses, we excluded points based on HDOP values, the number of satellites, and the distance to other points (*n* = 12 points removed; see S1 File for specific details on filtering). After all filtering, 91% of points used in home range analyses (n = 323/355) had HDOP values < 5 (good to ideal range; mean = 2.4, range = 0.8–18.4) and 84% had > 5 satellites (mean = 6 satellites, range = 3–9 satellites). Only eight remaining points had fair HDOP values (> 10) and seven had 2D fixes (3 satellites), with no points having both of these criteria (e.g., points with fair HDOP values had >3 satellites). These 15 points were also visually inspected and were not spatial outliers to clusters containing points with a high probability of accuracy (i.e., they were between 1–300 m from highest quality locations). Therefore, we assumed these points had high accuracy and retained them to bolster sample sizes for home range analyses.

### Home range area

There are a variety of methods for estimating home ranges, with minimum convex polygons (MCP) and kernel density estimates (KDE) being two of the most commonly used methods [7, 36]. MCPs draw the smallest possible boundary around outer points, and, therefore, are meant to represent a minimum area of use. MCPs are commonly used to make comparisons to previous studies, but they are recognized as unstable and subject to bias depending on sample size and the pattern of available points, potentially including large unused areas in the home range estimate [36]. However, as many previous studies have used MCPs, we created both KDEs and MCPs for each home range estimated. Additionally, because the number of locations available for territory estimation (see below) was not appropriate for KDEs, we use MCPs as a way to compare the relative space use determined through GPS tags and spot-mapping.

KDEs not only define a home range boundary, but also identify areas that are used disproportionately within the home range through application of a smoothing factor, or bandwidth (i.e., the distance over which a data point influences a utilization distribution [37, 38]). Home range estimates are sensitive to the bandwidth choice [36], and although least-squares cross-validation (LSCV) is commonly recommended for calculating an appropriate bandwidth [39], LSCV can produce high variability in bandwidths when there are a small number of points (e.g., <100) and is not recommended [40]. A typical alternative is to use the reference bandwidth [36]. The reference bandwidth has been shown to have the lowest absolute error for point patterns that reflect a “nest tree” behavior: a highly concentrated patch of points within a broader home range, as one might expect from a nesting bird that would forage away from the nest but return to that site often [41]. When using the reference bandwidth, home range sizes are also less sensitive than LSCV to sample size [39].

Home range estimates based on KDEs can be robust to sample sizes as low as 10 if points are spaced out temporally [42]. To determine the appropriate minimum number of points for our KDEs (fixed kernel method [39]), we created accumulation curves using code adapted from [43]. We created accumulation curves for any tags with at least 10 breeding points (n = 20; see S1 File for details). Based on the output from accumulation curves, we created fixed KDEs for tags with at least 13 points (n = 18), as this was where KDEs tended to stabilize (S1 Fig), and we used the reference bandwidth (calculated separately for each unique home range) with the *adehabitatHR* package in R [44]. Using 13 points is also similar to previous studies (e.g., house sparrow (*Passer domesticus*) using 20 points [45] and Swainson’s Warbler, using 15 points [11]). We calculated 95% KDEs, which we considered to represent the full home range and to include forays outside the core area, and 50% KDEs, which we considered to represent core-use areas. Two birds had two distinct clusters within their breeding range that were occupied sequentially (the switch for tag number 19388 occurred sometime between 25–30 July and for 81319 between 15–19 July); for these we calculated separate MCPs but a single KDE (when possible) to represent the full home range area. We used unscaled coordinates to create KDEs, unless the ratio of standard deviations of latitude and longitude was unequal (not near 1, i.e., <0.67 or >1.5; this occurred for six KDEs: tag numbers 49189, 49194, 49778, 49780, 49870, 81319), in which case we transformed the coordinates by dividing by the respective standard deviation [46]. For all tags with at least five points we created MCPs (n = 25); specifically, to account for potential infrequent movements or explorations outside the bird’s main area of use, we created 95% MCPs, following [47]. We used ArcGIS desktop 10.8.1 [48] to calculate the area for each KDE and MCP.

### Home range comparisons and characteristics

Land cover can be an important driver of home range size, with less suitable habitat potentially resulting in larger home ranges [49, 50]. At each home range, we determined land cover type at breeding grounds using the 2020 North America Land Change Monitoring System map [51]. We used the Zonal Histogram tool in ArcMap [48] to count the number of cells (30 m x 30 m) of each land cover class intersecting and/or completely within each 95% and 50% KDE. Using the centroids at each 50% KDE, we also determined latitude and used the *elevatr* package in R [52] to extract an elevation value at that point as a general representation of elevation in the home range. While terrain ruggedness index values are often used to determine the topographical range within an area and are calculated by comparing a center cell to eight neighboring cells [53], our home ranges varied in size and shape, so we instead used the *elevatr* package to determine the standard deviation of elevation in each home range as a measure of elevational heterogeneity.

To investigate whether latitude, elevation, sex, or the proportion of shrubland in the home range had an effect on the size of home ranges, we conducted a series of univariate linear regressions using the *stats* package in base R [54]. We ran univariate models because of low sample sizes and the recommendation to have 10–20 data points per predictor [55]. We used both 50% KDE area and 95% KDE area as the response variables. Our sample sizes were not large enough to include random effects in the models, so for birds tracked twice, we included the KDE that was created with the most GPS fixes so that each bird was represented only once. After initial regressions, we evaluated model residuals. To facilitate comparison across models, we removed outlier KDEs from all regressions when outliers were identified in any single regression (50% KDE outlier was 49202 and 95% KDE outliers included 49202, 49191, 81319, and 81324). We calculated the adjusted R^2^ and RMSE values of each regression using the *performance* package in R [56].

For the three birds that were tracked in two consecutive years, we determined the degree of overlap of the home range estimates. Specifically, we used the R package *amt* [57] to calculate the Bhattacharyya’s affinity between each set of utilization distributions per bird for both 95% and 50% KDEs. Bhattacharyya’s affinity is recognized as a formal method for comparing the degree of similarity between utilization distributions [58, 59], and it is a statistical measure of overlap ranging from 0–1, with zero representing no overlap and one representing 100% overlap [58].

### Home range timing

We obtained estimated arrival and departure dates at/from the breeding grounds for birds for which the start and end dates were no more than four days from available migration points (programming for tags was every two days on migration except for tag 19388 which was every five days). From these estimated dates, we present an estimated total duration (days) over which birds were on the breeding grounds. While exploring arrival dates, for some individuals, we found points showing the bird migrated to areas past their breeding area and then turned around to migrate in the opposite direction to reach the final home range, which could indicate reverse migration behavior. For those individuals, we estimated the distance and direction between the farthest spring migration points and the home ranges using the measure tool in ArcMap 10.8 (map projection North America Albers Equal Area Conic) for birds that migrated farther west than their breeding destination (Alaska breeding birds) or farther north than their breeding destination (British Columbia breeding bird).

### Territories measured by direct field observations

A subset of authors (E.J.H and D.S.) conducted field observations of Golden-crowned Sparrows at Hatcher Pass, Alaska (May-July 2012) and at Grey Mountain near Whitehorse, Yukon (May-July 2016). We captured territorial adults using mist nets paired with playbacks of conspecific songs. Individuals were sexed based on the presence of cloacal protuberance or brood patch, measured, and banded with a USGS metal leg band and a combination of three-color leg bands. We then opportunistically took GPS locations of banded birds during the nesting, incubation and nestling stages using a hand-held unit (Garmin GPSmap 64). We consider these points to represent territories as all points were taken when birds were perched on bushes, sometimes singing, and not while foraging. We obtained fewer than 13 points per bird, so we created 95% MCPs for these individuals and calculated the area. As there was no significant difference in territory area estimates between birds at Hatcher Pass (mean = 1.7 ha, *n* = 7) and Whitehorse (mean = 2.7 ha, *n* = 8; *t*(13.0) = -0.5, *p* = 0.6), we present summary statistics across all MCPs.

## Results

### Birds

Of the 50 birds GPS tagged at HAGM and UCD, one lost its tag before migration. Of the remaining 49, we successfully recaptured 29 birds (59%) the following fall/winter, three of which had lost their tag upon recapture. Four retrieved tags malfunctioned, resulting in 22 tags with data, including two tags each from three birds that were tracked twice. Of the tags with data, 21 tags had enough tracking data during the breeding season to determine a home range, including for both tags from each of the three birds tracked twice. At the UCSC site we retrieved five of the 30 tags in 2017 and one of the 40 tags in 2018 (9% across both years), likely due to human-induced alteration of a large portion of the winter study site within a month of tag deployment. Of the five tags retrieved in 2017 at UCSC, two malfunctioned leaving three with usable data, for a total of four tags with usable data from both years. For more details on the tag returns at UCSC, see [60]. This combined effort across field sites resulted in data from 23 individual birds, and 26 tags where three individuals were tagged and tracked across two seasons. The datasets generated and/or analyzed during the current study are available in the Movebank Data Repository for HAGM and UCD birds [61] and in the Dryad Repository for UCSC birds [62].

Sex determination for all individuals included 12 females (F) and 11 males (M): 4:10 F:M from UCD, 5:0 F:M from HAGM, and 3:1 F:M from UCSC (Table 1). For tags with at least five points captured on the breeding grounds, more points were recorded for males (mean = 20.4 points) than females (mean = 12.9 points; *F*(1, 23) = 7.48, *p* = 0.01, *n* = 25).

**Table 1:** Details on home ranges at breeding grounds for Golden-crowned Sparrows (*Zonotrichia atricapilla*) GPS-tagged at wintering grounds in California 2017–2020.

Tag	Bird	Sex	Age	Location Tagged	Date Tagged	Estimated dates at breeding grounds (month/day)	#of breeding pts for KDE/MCP	95% MCP size (ha)	95% KDE size (ha)	50% KDE (ha) /(# of core areas)
49209	A	F	SY	HAGM	1/4/2019	5/31 –NA	5*	1.1		
49218	B	F	SY	HAGM	1/4/2019	5/29 –[8/22]	11	9.0		
49222	C	F	SY	HAGM	1/4/2019	[6/11]–[9/12]	14	4.9	26.0	6.7 (1)
49201	D	F	AHY	HAGM	1/11/2019	[6/11]–[8/13]	8	27.1		
49200	E	F	SY	HAGM	1/11/2019	5/23 –NA	7*	0.03		
49206	F	F	AHY	UCD	2/19/2019	5/25 –[8/22]	14	1.8	8.7	1.8 (1)
49771	G	F	SY	UCD	2/4/2020	5/23 –NA	5*	0.2		
49775	H	F	SY	UCD	2/6/2020	5/29 –[8/15]	7	8.5		
49191	I	M	ASY	UCD	2/19/2019	[5/17]– 9/2	18	8.3	27.2	6.5 (1)
49192	J	M	SY	UCD	2/19/2019	**5/21–9/8**	21	9.1	34.8	9.4 (1)
49194	K	M	SY	UCD	2/20/2019	5/19 –[8/22]	16	1.9	22.6	3.5 (1)
49205	L	M	ASY	UCD	2/19/2019	5/19 –NA	4*	NA		
49217	M	M	ASY	UCD	2/19/2019	**5/23–9/4**	18	2.6	7.6	1.7 (1)
49777	N	M	AHY	UCD	2/4/2020	**5/17–9/6**	16	1.6	7.2	1.3 (1)
49780	O	M	SY	UCD	2/4/2020	**5/21–9/2**	14	2.4	11.4	2.5 (1)
49870	P	M	ASY	UCD	2/4/2020	**5/11–9/12**	21	6.7	26.3	6.6 (2)
49189	Q	F	ASY	UCD	2/20/2019	5/19 –[8/22]	17	1.7	9.6	2.2 (1)
49776	Q		ASY	UCD	2/6/2020	[5/17]– 8/31	13	2.2	11.2	3.0 (1)
49195	R	M	SY	UCD	2/19/2019	5/21 –[8/22]	16	25.2	142.2	22.9 (1)
49770	R		AHY	UCD	2/4/2020	**5/9–9/8**	20	4.5	17.5	3.3 (1)
49202	S	M	ASY	UCD	2/20/2019	**5/11–9/12**	26	12.1	91.3	12.7 (1)
49778	S		ASY	UCD	2/4/2020	**5/13–9/16**	22	36.7	283.9	60.9 (1)
19388	T	F	ASY	UCSC	1/17/18	[7/5]–NA	11*	1.1 / 0.4		
77968	U	M	ASY	UCSC	3/22/17	5/25 –[9/8]	36	4.1	10.2	2.2 (1)
81319	V	F	AHY	UCSC	3/20/17	**6/21–9/2**	21	1.2 / 0.4	44.6	8.9 (2)
81324	W	F	ASY	UCSC	3/19/17	**5/31–9/5**	32	2.0	11.0	1.8 (1)

Estimated home range sizes on breeding grounds for Golden-crowned Sparrows (*Zonotrichia atricapilla*) GPS-tagged at wintering grounds in California 2017–2020, along with individual bird information including sex (determined genetically), age (SY = second year, AHY = after hatch year, ASY = after second year), tagging location, and date tagged (month/day/year). Birds that were tagged twice are indicated in the Bird column by sharing the same letter but different tag numbers. The estimated dates at breeding grounds are based on points retrieved at breeding grounds. For birds that stopped tracking before fall migration, the end date at breeding grounds is given as NA. Dates with > 4 days of uncertainty between points on breeding grounds and spring migration (first date) or fall migration (last date) are in brackets, and indicate the birds for which we did not calculate a duration on breeding grounds. Dates that were used for calculating duration at breeding grounds are indicated in bold. KDE = kernel density estimate and MCP = minimum convex polygon; UCD = University of California, Davis; HAGM = Hagmaier Ranch, Olema Valley, Point Reyes National Seashore; UCSC = University of California, Santa Cruz.

*These tags stopped collecting points before the bird left the breeding grounds (no fall migration points).

### Home range areas from GPS tags

**Fig 2:**
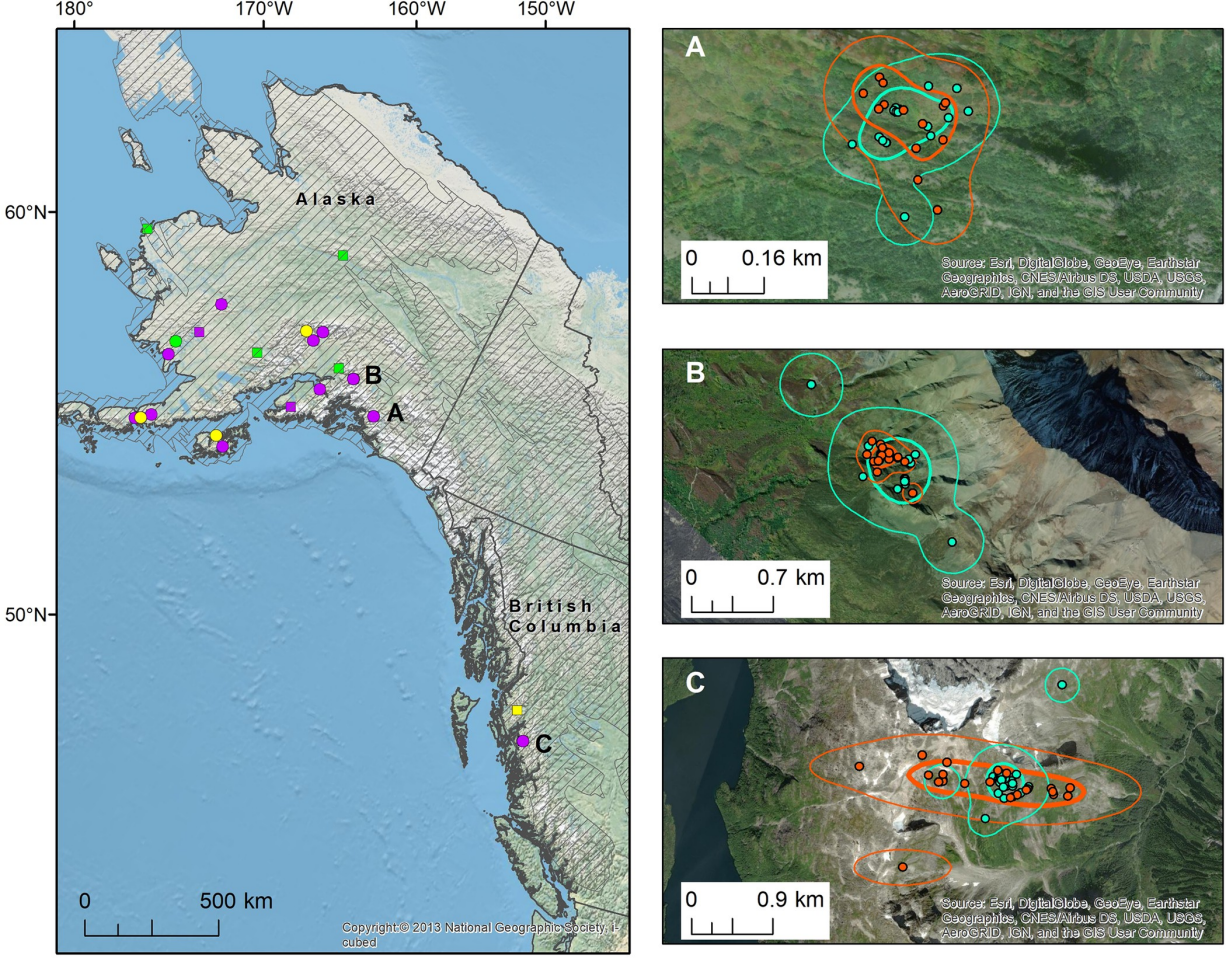
Golden-crowned Sparrow (*Zonotrichia atricapilla*) home ranges. Left panel: home ranges on breeding grounds in Alaska and British Columbia for Golden-crowned Sparrows GPS-tagged at wintering grounds in California 2017–2020. Shaded areas with gray hatching show portions of the breeding range (range map image provided by eBird (www.ebird.org) and created 9 May 2024). Purple represents birds tagged at the University of California Davis, green represents birds tagged at Hagmaier Ranch in Point Reyes National Seashore, and yellow represents birds tagged at University of California Santa Cruz. Circles represent home ranges that had enough points for kernel density estimates (KDEs), squares for minimum convex polygons (MCPs). Right side panels show repeat home ranges for three birds tracked twice, with letters corresponding to the location on the full map (left panel): Panel A is for Bird Q (female, tags 49189 and 49776), Panel B is for Bird R (male, tags 49195 and 49770), and Panel C is for Bird S (male, tags 49202 and 49778). Each GPS fix (point) is represented as a solid circle. The 50% KDEs are the inner (bolder) lines and 95% KDEs are the outer lines (see S2 Fig for KDEs from other tags). The turquoise color is for 2019 points and corresponding home ranges, and orange is 2020 points and home ranges.

Breeding destinations were in Alaska for all but two birds (one from UCD, one from UCSC), which had destinations in British Columbia (see S1 Table for centroid latitude and longitude), and the three birds that were tracked twice showed high site fidelity to their previous breeding areas (Fig 2). The number of retained points used in home range analyses ranged from 5–36 (mean ± SD = 16.4 ± 7.7 points; Table 1). From this, we calculated 18 KDEs for 15 birds; the 95% KDEs representing home ranges ranged in size from 7.2–283.9 ha (mean ± SD = 44.1 ± 69.1 ha). The 50% KDEs, representing the core area of use, ranged from 1.3–60.9 ha (mean ± SD = 8.8 ± 14.1 ha; Table 1 and S2 Fig). We calculated 27 95% MCPs (for 25 tags because we calculated two separate MCPs for two tags) with GPS tag data, ranging in size from 0.03–36.7 ha (mean ± SD = 6.5 ± 9.1 ha; Table 1 and S2 Fig).

### Home range comparisons and characteristics

**Fig 3:**
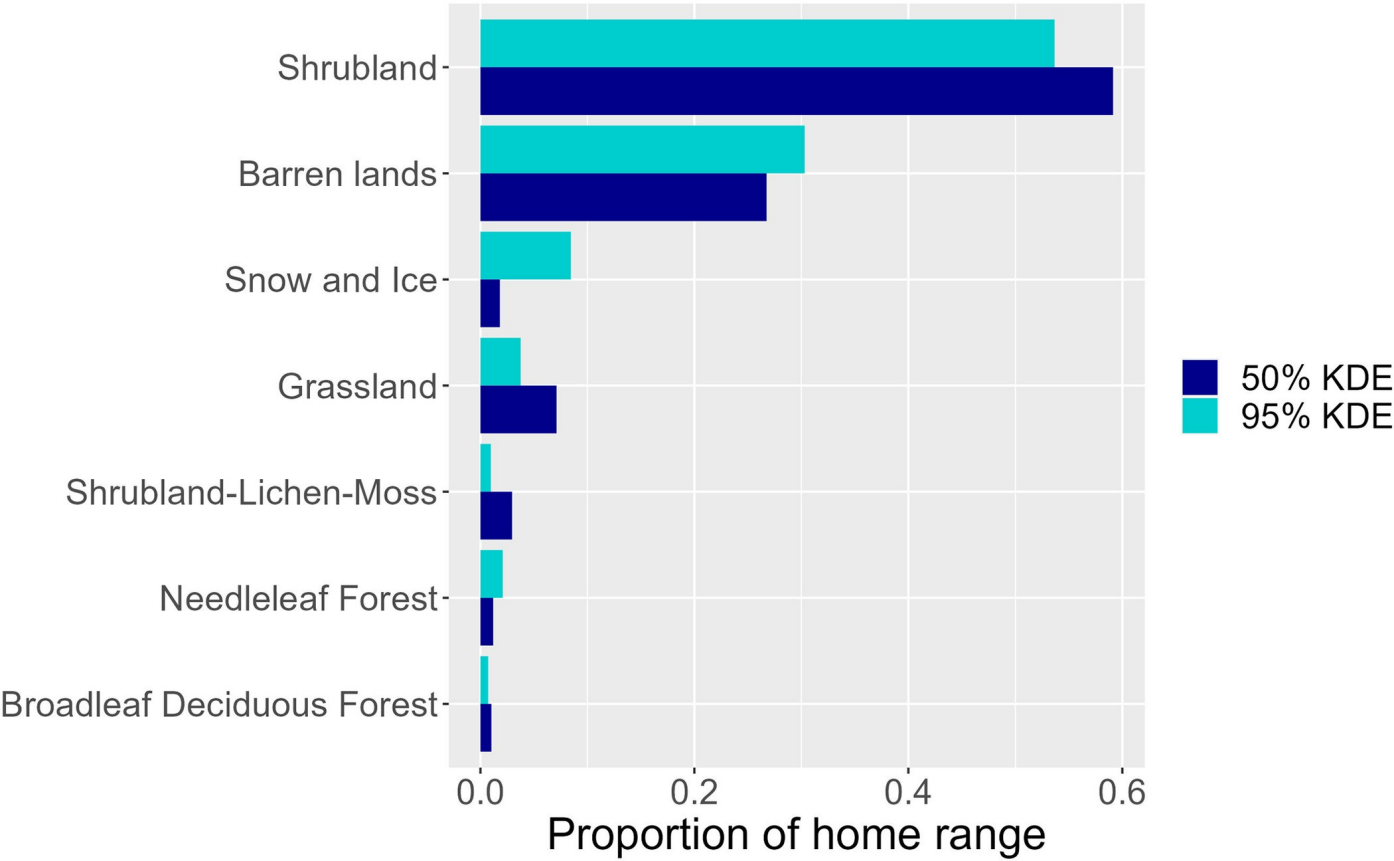
The proportion of land cover classes at home ranges on breeding grounds in Alaska and British Columbia for Golden-crowned Sparrows (*Zonotrichia atricapilla*) GPS-tagged at wintering grounds in California 2017–2020, for all KDEs combined. Land cover classes are from the 2020 North America Land Change Monitoring System map (NALCMS 2023). Shrubland = “Temperate or sub-polar shrubland”; Grassland = “Temperate or sub-polar grassland”; Shrubland-lichen-moss = “Sub-polar or polar shrubland-lichen-moss”; Needleleaf forest = “Temperate or sub-polar needleleaf forest”; Broadleaf deciduous forest = “Temperate or sub-polar broadleaf deciduous forest”.

Land cover classes at home ranges included at least some shrubland for all birds (Fig 3), and shrubland was the only land cover class included in every home range and core area (S2 Table). Twelve of the 18 core areas and 10 of the overall home ranges included 90% or more shrubland land cover (10 core areas and seven home range had 100% shrubland cover), and all but one bird (Bird S, tags 49202 and 49778) had >57% shrubland land cover at their core areas (and >64% for overall home ranges). The second most common land cover was barren lands, but this was driven primarily by Bird S where approximately half of the KDEs had barren land (S2 Table and S2 Fig), and this land cover class was either not present in most home ranges or did not constitute a large percent of the home range (less than 8% for three 50% KDEs, and less than 18% for six 95% KDEs). Shrubland was one of the most common available habitats near home ranges (see S3 Fig). Home ranges also included other land cover classes including snow/ice, grassland, shrubland-lichen-moss, needleleaf forest, and broadleaf deciduous forest (Fig 3).

Elevation at KDE centroids ranged from 24–1408 m (mean ± SD = 664 ± 446 m). The standard deviation of elevation values, representing elevational heterogeneity, at 95% KDEs ranged from 0.97 m to 283.4 m and 50% KDEs ranged from 0.78 m to 122.9 m. After removing outliers, 50% KDE regression models included 15 KDEs and 95% KDE models had 11 KDEs. The R^2^ values were 0.2 or less for most models, indicating that latitude, the proportion of shrubland, and sex were not important predictors of home range size (Table 2). The univariate model with the highest adjusted R^2^ value was the elevational heterogeneity model, for both 50% KDEs (adjusted R^2^ = 0.532, *F*(1, 12) = 15.77, *p* < 0.01) and 95% KDEs (adjusted R^2^ = 0.386, *F*(1, 9) = 7.299, *p* < 0.5). In both cases, home range size increased as elevational heterogeneity increased, though this effect was stronger for 50% KDEs (with 1 ha increase in home range size, there was an increase of 0.10 in the standard deviation of elevation values (*t*(12) = 3.97, *p* < 0.01, *β* = 0.10, 95% CI [0.05, 0.16]) than for 95% KDEs (with 1 ha increase in home range size, there was an increase of 0.27 in the standard deviation of elevation values (*t*(9) = 2.70, *p* <0.05, *β* = 0.27, 95% CI [0.04, 0.50]).

**Table 2:** Linear regression models for effects on home range size for Golden-crowned Sparrows.

Predictor	50% KDE		95% KDE	
	Adj R^2^	RMSE	Adj R^2^	RMSE
Elevation Standard Deviation	0.532	1.803	0.386	6.730
Latitude	0.205	2.350	0.198	7.694
Centroid Elevation	0.125	2.465	0.090	8.195
Proportion Shrubland	-0.080	2.739	-0.074	8.902
Sex	-0.082	2.742	-0.095	8.990

Linear regression models for effects on home range size (50% KDE and 95% KDE) for Golden-crowned Sparrows (*Zonotrichia atricapilla*) at breeding grounds that were GPS-tagged at wintering grounds in California 2017–2020, with adjusted R^2^ (Adj R^2^) and root mean square error (RMSE) values. All models for 50% KDEs and all models for 95% KDEs included the same datasets (i.e., same outliers were removed, see Methods). For birds tracked twice, only one home range value per bird was used.

Using the Bhattacharyya’s affinity measure, we determined that the degree of similarity between core areas (50% KDEs) for birds tagged twice was 0.664, 0.368, and 0.373 for Birds Q (female), R, and S (both males) respectively. The degree of similarity between the overall home ranges (95% KDEs) was 0.829, 0.529, and 0.538 for Birds Q, R, and S respectively.

### Home range timing

Males arrived at breeding grounds on average nine days earlier (mean = 20 May, *n* = 12) than females (mean = 29 May, *n* = 9). Total duration in days at breeding grounds was on average 110 days (SD: 16.3 days, range = 73–126 days, *n* = 10; see dates bolded in Table 1 for those that met the criteria for calculating total duration). Only three females met our criteria for calculating departure dates, with an average breeding departure of 3 Sept (range = 31 Aug-5 Sept, n = 3); however, the latest known point on the breeding grounds for another female (with greater uncertainty in actual departure date) was 12 Sept. Males departed on average on 8 Sept (range = 2 Sept-16 Sept, *n* = 9); given these data, males stayed on breeding grounds on average five days longer than females across the entirety of the season. However, we did not statistically test for differences in migration timing between the sexes due to the uncertainty in arrival and departure dates.

We found evidence that five GPS-tagged birds migrated past their final destination before turning to migrate in a different direction (S4 Fig). In three cases, the migration points before turning back (representing the farthest they traveled) were a relatively small distance from the home range, and these distances include the following: 9.6 km to the NW for tag 49195, 7 km to the NE for tag 49778 (British Columbia breeding birds), and 21 km to the NW for tag 49771. In two cases, the distances were larger. For tag 49206, the last spring migration point was 65 km to the SW of the home range and for tag 49201, there were two points to the NW of the home range: one 154 km away on 31 May, then another 74 km away on 2 June, as the bird appeared to be making its way “back” to the breeding area.

### Territory sizes measured by direct field observations

We created 15 95% MCPs from direct observations of marked individuals in the field. This included seven birds from Whitehorse, Yukon in 2017 and eight birds from Hatcher Pass, Alaska in 2012 (5–12 GPS locations per bird, mean ± SD = 8.7 ± 2.34 locations). The territories ranged in size from 0.09 ha to 10.9 ha (mean ± SD = 2.1 ± 3.4 ha, *n* = 15). For both the average and maximum 95% MCP areas, territory sizes measured from these direct observations were on average approximately three times smaller than those created with GPS tags.

## Discussion

We provide new knowledge of space use behavior during the breeding season and immediately before arrival to breeding areas for the Golden-crowned Sparrow, the only *Zonotrichia* species without extensive detailed data available for home ranges on the breeding grounds. With GPS tags deployed on Golden-crowned Sparrows on their wintering grounds in California, we documented home ranges for both sexes in their breeding range in Alaska and British Columbia, and tracked three of these birds twice.

### Home range and territory area

We documented much larger space use with miniaturized GPS tags carried by the birds than from territory estimates based on field observations, indicating that Golden-crowned Sparrows move relatively large distances on the breeding grounds that are not being detected in the field by spot-mapping. Similar space use dynamics may be occurring for other passerine species for which there have only been territory estimates in the field (as also supported by recent radio telemetry studies [15, 16]), and GPS tracking offers a chance to discover larger and more secretive movements—including, as in this study, by tagging birds on their wintering grounds and passively tracking their home range movements on their breeding grounds.

Little quantitative data exist on Golden-crowned Sparrow territory or overall home range size. The territory size estimates we report for Golden-crowned Sparrows from mapping territories in the field (2.1 ha on average) are larger than what has been found for this species previously (average nesting area in shrub thickets was 0.012 ha, and territory size in British Columbia was estimated at 1 ha [27]). Similarly, for other *Zonotrichia* species, reported territory estimates have all been less than 1 ha (ranging from 0.1 ha to 0.79 ha for non-migratory White-crowned Sparrows, White-throated Sparrows, and Rufous-collared Sparrow *Zonotrichia capensis* [63–67]). All of these estimates for the other *Zonotrichia* species, and for Golden-crowned Sparrow territory size as part of this study, were based on identifying individual birds (generally color-banded) in the field without using external tracking devices. When other *Zonotrichia* species were tracked with external tracking devices, their home range estimates were larger than the 1 ha maximum territory sizes described above. For example, radio telemetry of Mountain White-crowned Sparrows (*Z*. *l*. *oriantha*) breeding in the central Sierra Nevada of California revealed home range 95% MCPs from 0.4 ha to 11.4 ha [68] and GPS tracking of birds in that same area (n = 17 for four days each) revealed 95% MCPs up to 13.5 ha (C. Hawkins, unpubl. data). Our estimates of home ranges for Golden-crowned Sparrows based on GPS tags are considerably larger (44.1 ha for 95% KDE).

There are a variety of potential reasons for the larger home ranges we found for Golden-crowned Sparrows compared to the previous studies listed above. It may be the different utilization distribution approach (KDE instead of MCP). However, when comparing our 95% MCPs to the aforementioned studies using external tracking devices, our mean value (6.5 ha) aligns with the prior results, but our maximum MCP size was about three times larger (up to 36.7 ha). It is also possible that habitat quality, and potentially breeding bird density, differs between sites where territories have been measured for Golden-crowned Sparrows and sites with home ranges estimated through GPS tagging. Birds may also be expanding their space use after breeding when they enter the molting phase once breeding is complete, which can sometimes occur in a completely different area, or at the edges of the already established territory [69, 70]; it is possible the secondary home range we observed in mid to late July were due to moving to an area to molt. However, throughout the breeding season, the distance from each location to the centroid did not increase overall as the season progressed (S5 Fig). Future studies linking movements through tracking (with a higher frequency of locations) combined with trapping to assign life stages would help us understand how Golden-crowned Sparrow movements may shift seasonally.

We filtered points conservatively in line with manufacturer recommendations and previous studies (that found points with high HDOP and 3 satellites should be down weighted [50]) and expect a high accuracy for our locations. However, further studies could improve the accuracy of home range estimates and determine how space use changes throughout the breeding season by collecting more points on the breeding grounds as tracking technology improves and/or through individual tag calibration to assess tag-specific error that can be incorporated into modeling [50, 71]. More generally, our data align with other studies that show how visual and auditory observations of breeding birds have the potential to miss more subtle movements outside of territories [15, 16, 19]. Likewise, researchers may even intentionally restrict the data used to create estimated territory boundaries, as such methods (e.g., spot mapping; [10]) are typically used to estimate the core breeding territory and not the broader home range, and territory estimates based on singing may intentionally exclude areas where other birds sing even if home range overlaps may be occurring. Our study supports that territories and home ranges should not be conflated.

### Home range comparisons and characteristics

We found that larger elevational heterogeneity correlated with larger home range sizes for Golden-crowned Sparrows. As we did not calculate a three-dimensional surface area for each home range this effect may even be stronger, and actual home ranges larger, than we estimated, as an increase in elevational variation would also increase the surface area available to animals [72]. A greater elevational heterogeneity predicted smaller home ranges for Golden Eagles (*Aquila chrysaetos*) presumably due to an increased amount of suitable habitat and prey [73], and so it is possible that for Golden-crowned Sparrows, larger elevational heterogeneity provides less suitable habitat, which results in larger home ranges. This could be due to steeper slopes providing less suitable or lower quality shrub habitat, and/or a separation of high-quality nesting and foraging habitat in higher elevational areas with more barren land and snow. While we did not find the proportion of shrubland to be a very strong predictor for home range size, it should be noted that shrubland cover was also measured two-dimensionally and so its importance may have been underestimated. Also, we did not have confirmation that sparrows bred successfully on their home ranges, and larger home ranges could represent birds that did not successfully breed and became “floaters” over habitat less suitable for nesting [74].

Our dataset included more points from males than females. Female Golden-crowned Sparrows build the nest, incubate eggs and brood nestlings while males do not; one female in another study was reported spending 72% of her time incubating and up to 67% of the day on the nest when brooding began (with sex presumed based on crown [75]). As Golden-crowned Sparrows nest on or very near the ground, and often inside shrubs [75, 76], likely the line of sight to satellites was reduced for females during the breeding season, resulting in fewer GPS points for females. Males sing from exposed perches during the breeding season [27], which could have increased their chances of being picked up by satellites during the morning hours when the tags were set to collect data. To increase satellite fixes, we recommend considering when birds may be more likely incubating (e.g., during cold morning hours) when programming tags. Despite females having fewer points, sex was not an important predictor for home range size. Additionally, males may have had more points on the breeding grounds because they spend more time there, as we found that males arrived on average nine days earlier than females (although our result about departure timing by sex was equivocal). Previous publications on Golden-crowned Sparrows show arrival on the breeding grounds in May in British Columbia and Alaska consistent with our findings, but the difference in arrival times between males and females has been poorly documented [27]. The earlier arrival at the breeding grounds by males we found is consistent with the potential evolutionary advantage to earlier male arrivals, likely acting to increase mating opportunities through earlier territory establishment [77]. As tag technology improves for small birds, it may become possible to collect more fine-scale data points to determine arrival (and departure) dates more precisely, along with migration tracks.

Our data support that shrub habitat is important for breeding in this species (as was previously described [27]), with shrubland cover the only habitat type present in every home range (KDEs), and, for most home ranges, the dominant land cover. However, we found that home ranges for some individuals included a variety of other habitat types (consisting especially of open habitats such as barren lands, and to a much lesser extent forested habitats), which is also consistent with general descriptions of the breeding range [27]). Although there are no quantitative data available on the characteristics of nest sites, nests have been found in shrub habitats and at the edges of open woodlands, on the ground, in shrubs, or sometimes in small trees, willows, or dwarf conifers ([27]; Shizuka and Hudson, unpublished data). It is unknown whether a mix of habitats with a variety of cover and resources, versus true shrubland habitat, provides more ideal territories or influences breeding success. We did not find the proportion of shrubland within the home range to be an important predictor for home range size, but the maximum size of 95% KDEs with 100% shrub cover was only 34.8 ha, which is slightly smaller than the mean 95% KDE size across all birds (44.1 ha). This could suggest that shrub habitat is of higher quality so that less area is needed for a breeding pair [49, 50], or that habitat quality correlates with population density [78] which restricts the size of an area that can be defended. Due to the limited sample size (18 KDEs), this requires further study and would benefit from fitness estimates in the field.

### Site fidelity

We found high breeding site fidelity for birds tracked in more than one year. Each of the three birds (1 female and 2 males) returned to approximately the same breeding territory in both years they were tracked. This is consistent with previous findings for Golden-crowned Sparrows banded in the 1930s that returned to the same breeding areas in multiple years [79]. Similarly, Golden-crowned Sparrows also have very high site fidelity to their winter home ranges [80, 81], where the sparrows form loose foraging flocks and have been shown to socialize with the same individuals across multiple years [82, 83]. However, we also found a degree of flexibility at breeding sites, as two birds had two distinct clusters within their breeding range that they occupied sequentially in the same year, indicating a shift in home range or territory. It is possible that these reflect spatially disjunct sequential nesting attempts (for example, if the first nest site failed and the resulting renesting location required a territory shift), a within-season change in mate, or movements due to molting.

### Reverse migration

We found that for some birds, arrival to home ranges at the breeding grounds showed interesting movement paths in the form of reverse migration. Birds will sometimes migrate in the reverse direction of what is expected for the season; reasons given for this reverse migration vary and have included wind and cloud cover, birds seeking out suitable resting locations, and bird body condition, among others [84–86]. Juvenile passerines may also be more likely to fly in a reverse direction (opposite to what is seasonally appropriate) than adults [86, 87]. We found that some Golden-crowned Sparrows displayed short-distance “reverse migration” in the spring, related to apparent corrections in their path or potentially as a response to environmental conditions along the route. Specifically, some birds migrated farther west (for Alaska breeders) or north (for British Columbia breeders) than their final home range area and reversed (or in some cases, significantly changed) course before settling on the breeding grounds. We do not know the body condition for these birds at the time they turned around, or the active weather conditions they may have been encountering when they changed course. One bird migrated at least 154 km to the northwest of the home range and flew back over the course of a few days. Reverse migration has not often been reported for New World Sparrows on spring migration (nor adequately studied), but White-crowned Sparrows that were experimentally displaced longitudinally in the Arctic reversed their migration direction to reach their original breeding grounds [88]. Additionally, White-throated Sparrows with low fat reserves during migration have been shown to vary their orientation away from what is seasonally expected [89], and juvenile Ipswich Savannah Sparrows (*Passerculus sandwichensis princeps*) displayed some reverse migration during fall [87].

## Conclusion

Despite decades of research, the distinction between home range and territory remains blurry. This is in part due to the dynamic nature of spatial behavior that reflects the social and ecological aspects of territoriality (e.g., resource defense, courtship) and maintenance of home ranges (foraging, information collection, solicitation of extra-pair matings). The combination of field observations and technological advancement can be combined to reveal previously hidden dimensions of spatial behavior. For example, Acorn Woodpeckers (*Melanerpes formicivorus*) vigorously defend group territories where they store resources (acorn granaries), yet automated radiotelemetry methods reveal that individuals make daily forays of 500–600 m to monitor territories held by other social groups [90]. Similarly, automated telemetry of Kirtland’s Warblers revealed that many non-breeders and some breeders made large-distance movements during the breeding season (5–77 km), often at night, presumably to gather information to inform dispersal in subsequent years [20]. Field observations and experimental studies of Golden-crowned Sparrow males show that they defend territories through singing and chasing other courting males [27, 91]. However, the current study shows that territory size estimates based on field observations do not match the scale of movements of individuals during the breeding season as revealed by miniature GPS tags. Further work that combines field observation with technologies such as GPS tagging or automated telemetry have the potential to reveal the behavioral and functional basis of differences in territorial behavior and larger home-range spatial movements, including for within-season changes in life stages such as molt, and to investigate how territory establishment may not be exclusive of overlapping space use in home ranges. Such efforts have the potential to enhance our knowledge of both common and poorly studied species, and, by applying remote technologies like that done in this study, are sure to produce critical insights into species that migrate or occur in remote areas that may be difficult for researchers to access, and whose life history in other portions of their annual cycle may otherwise remain a mystery. By understanding space use on the breeding grounds with more precision, and understanding the habitats that support various life stages, conservation management can more effectively protect important habitat, a critical step in preventing or reversing population declines.

## Supporting information

S1 Fig Accumulation curves for Golden-crowned Sparrow breeding locations and home range size (50% KDEs and 95% KDEs), for Golden-crowned Sparrows (Zonotrichia atricapilla) GPS-tagged at wintering grounds in California 2017–2020. Each set of GPS points for each bird (with at least 10 points) was randomly sampled 20 times at various sample sizes (from five to the total points available), and KDEs were created at each sample size. From this, we decided on a cutoff of 13 points for creating home range estimates. Each curve title refers to the tag number.
(PDF)

S2 Fig Filtered breeding locations in Alaska and British Columbia and associated home range estimates for Golden-crowned Sparrows (Zonotrichia atricapilla) GPS-tagged at wintering grounds in California 2017–2020. Home range estimates include kernel density estimates (KDEs; given as blue polygons with darker shading indicating 50% KDEs and lighter shading 95% KDEs) and minimum convex polygons (MCPs; given as yellow shapes).
(PDF)

S3 Fig Land cover classes in Alaska. Throughout the breeding area of Golden-crowned Sparrows GPS-tagged at wintering grounds in California 2017–2020, shrubland was widely available. This figure shows a close-up of breeding centroids (pink circles) in Alaska, and shrubland areas are shown in brown tones (land cover classes 7, 8, and 11). The full land cover names for this layer are as follows (not all possible types are shown in this map): 1 = Temperate or sub-polar needleleaf forest; 2 = Sub-polar taiga needleleaf forest; 3 = Tropical or sub-tropical broadleaf evergreen forest; 4 = Tropical or sub-tropical broadleaf deciduous forest; 5 = Tropical or sub-polar broadleaf deciduous forest; 6 = Mixed forest; 7 = Tropical or sub-tropical shrubland; 8 = Temperate or sub-polar shrubland; 9 = Tropical or sub-tropical grassland; 10 = Temperate or sub-polar grassland; 11 = Sub-polar or polar shrubland-lichen-moss; 12 = Sub-polar or polar grassland-lichen-moss; 13 = Sub-polar or polar barren-lichen-moss; 14 = Wetland; 15 = Cropland; 16 = Barren land; 17 = Urban and built up; 18 = Water; 19 = Snow and ice.
(PDF)

S4 Fig Reverse migration for Golden-crowned Sparrows (Zonotrichia atricapilla) GPS-tagged at wintering grounds in California 2017–2020. Five birds showed reverse migration, flying in the opposite direction expected for migration (i.e. south or west). Yellow dots and polygons represent final destinations (centroids, and home ranges where visible). Green points are migration locations and are connected by a purple line to demonstrate directionality for the birds.
(PDF)

S5 Fig The distance to centroids (created from kernel density estimates [KDEs]) from each location (date given on x axis for each location) on the breeding grounds for Golden-crowned Sparrows (Zonotrichia atricapilla) GPS-tagged at wintering grounds in California from 2017–2020. This figure shows 355 points across 19 KDEs (see Table 1 for details on all KDEs).
(PDF)

S1 Table Home range center points (centroids of 50% KDEs) for Golden-crowned Sparrows (Zonotrichia atricapilla) GPS-tagged at wintering grounds in California 2017–2020. For two tags, there were two core areas and a center point is given for each.
(PDF)

S2 Table Land cover classes (the number of cells 30m x 30m) represented in each home range (50% and 95% KDEs), along with the proportion of shrubland represented across all cells, for Golden-crowned Sparrows (Zonotrichia atricapilla) GPS-tagged at wintering grounds in California 2017–2020. Land cover classes are from the 2020 North America Land Change Monitoring System map (NALCMS 2023). Needleleaf forest = “Temperate or sub-polar needleleaf forest”; Broadleaf forest = “Temperate or sub-polar broadleaf deciduous forest”; Shrubland = “Temperate or sub-polar shrubland”; Grassland = “Temperate of sub-polar grassland”; Shrubland-lichen-moss = “Sub-polar or polar shrubland-lichen-moss”.
(PDF)

S1 File Supplemental methods. (PDF)

## References

[1] Nathan R, Getz WM, Revilla E, Holyoak M, Kadmon R, Saltz D, et al. A movement ecology paradigm for unifying organismal movement research. Proc Natl Acad Sci. 2008;105: 19052–19059. doi: 10.1073/pnas.0800375105 19060196 PMC2614714

[2] Katzner TE, Arlettaz R. Evaluating Contributions of Recent Tracking-Based Animal Movement Ecology to Conservation Management. Front Ecol Evol. 2020;7. doi: 10.3389/fevo.2019.00519

[3] Maher CR, Lott DF. Definitions of territoriality used in the study of variation in vertebrate spacing systems. Anim Behav. 1995;49: 1581–1597. doi: 10.1016/0003-3472(95)90080-2

[4] Giuggioli L, Kenkre VM. Consequences of animal interactions on their dynamics: Emergence of home ranges and territoriality. Mov Ecol. 2014;2: 1–22. doi: 10.1186/s40462-014-0020-7 25709829 PMC4337768

[5] Burt WH. Territoriality and Home Range Concepts as Applied to Mammals. J Mammal. 1943;24: 346–352.

[6] Powell RA. Animal home ranges and territories and home range estimators. In: Boitani L, Fuller T, editors. Research techniques in animal ecology: controversies and consequences. New York: Columbia University Press; 2000. pp. 65–110.

[7] Laver PN, Kelly MJ. A Critical Review of Home Range Studies. J Wildl Manage. 2008;72: 290–298. doi: 10.2193/2005-589

[8] Nice MM. The Role of Territory in Bird Life. Am Midl Nat. 1941;26: 441. doi: 10.2307/2420732

[9] Börger L, Dalziel BD, Fryxell JM. Are there general mechanisms of animal home range behaviour? A review and prospects for future research. Ecol Lett. 2008;11: 637–650. doi: 10.1111/j.1461-0248.2008.01182.x 18400017

[10] Ralph CJ, Geupel GR, Pyle P, Martin TE, DeSante DF. Handbook of field methods for monitoring landbirds. General Technical Report PSW-GTR-144. Albany; 1993.

[11] Anich NM, Benson TJ, Bednarz JC. Estimating territory and home-range sizes: Do singing locations alone provide an accurate estimate of space use? Auk. 2009;126: 626–634. doi: 10.1525/auk.2009.08219

[12] Alcock J. Avian Mating and Social Behavior. In: Lovette IJ, Fitzpatrick JW, editors. The Cornell Lab of Ornithology Handbook of Bird Biology. West Sussex: John Wiley and Sons Inc.; 2016. pp. 313–354.

[13] Neudorf DL, Stutchbury BJM, Piper WH. Covert extraterritorial behavior of female hooded warblers. Behav Ecol. 1997;8: 595–600. doi: 10.1093/beheco/8.6.595

[14] Ryan Norris D, Stutchbury BJM. Extraterritorial movements of a forest songbird in a fragmented landscape. Conserv Biol. 2001;15: 729–736. doi: 10.1046/j.1523-1739.2001.015003729.x

[15] Connare B, Islam K. Advancing our understanding of Cerulean Warbler space use through radio telemetry. J Fish Wildl Manag. 2023;14: 75–89.

[16] Frantz MW, Aldinger KR, Wood PB, Duchamp J, Nuttle T, Vitz A, et al. Space and habitat use of breeding Golden-winged Warblers in the Central Appalachian Mountains. In: Streby HM, Andersen DE, Buehler DA, editors. Golden-winged Warbler ecology, conservation, and habitat management Studies in Avian Biology (no 49). Boca Raton: CRC Press; 2016. pp. 81–94.

[17] Naguib M, Altenkamp R, Griessmann B. Nightingales in space: song and extra-territorial forays of radio tagged song birds. J für Ornithol. 2001;142: 306–312. doi: 10.1046/j.1439-0361.2001.01005.x

[18] Brouwer L, Griffith SC. Extra-pair paternity in birds. Mol Ecol. 2019;28: 4864–4882. doi: 10.1111/mec.15259 31587397 PMC6899757

[19] Hanski IK, Haila Y. Singing territories and home ranges of breeding Chaffinches: visual observation vs. radio-tracking. Ornis Fenn. 1988;65: 97–103.

[20] Cooper NW, Marra PP. Hidden Long-Distance Movements by a Migratory Bird. Curr Biol. 2020;30: 4056–4062.e3. doi: 10.1016/j.cub.2020.07.056 32822609

[21] Bowler DE, Heldbjerg H, Fox AD, de Jong M, Böhning-Gaese K. Long-term declines of European insectivorous bird populations and potential causes. Conserv Biol. 2019;33: 1120–1130. doi: 10.1111/cobi.13307 30912605

[22] Rosenberg K V., Dokter AM, Blancher PJ, Sauer JR, Smith AC, Smith PA, et al. Decline of the North American avifauna. Science (80-). 2019;366: 120–124. doi: 10.1126/science.aaw1313 31604313

[23] Iverson AR, Schaefer JLB, Skalos SM, Hawkins CE. Global positioning system (GPS) and platform transmitter terminal (PTT) tags reveal fine-scale migratory movements of small birds: A review highlights further opportunities for hypothesis-driven research. Ornithol Appl. 2023;125: 1–16. doi: 10.1093/ornithapp/duad014

[24] Campion D, Pardo I, Elósegui M, Villanua D. GPS telemetry and home range of the White-backed Woodpecker Dendrocopos leucotos: Results of the first experience. Acta Ornithol. 2020;55: 77–87. doi: 10.3161/00016454AO2020.55.1.008

[25] Musseau R, Bastianelli M, Bely C, Rousselle C, Dehorter O. Using miniaturized GPS archival tags to assess home range features of a small plunge-diving bird: the European Kingfisher (Alcedo atthis). Avian Res. 2021;12: 1–10. doi: 10.1186/s40657-021-00267-4

[26] Cadieux P, Boulanger Y, Cyr D, Taylor AR, Price DT, Sólymos P, et al. Projected effects of climate change on boreal bird community accentuated by anthropogenic disturbances in western boreal forest, Canada. Divers Distrib. 2020;26: 668–682. doi: 10.1111/ddi.13057

[27] Norment CJ, Hendricks P, Santonocito R. Golden-crowned Sparrow (*Zonotrichia atricapilla*). In: Birds of the World [Internet]. 2020. Available: https://doi.org/10.2173.bow.gocspa.01

[28] Pyle P. Identification Guide to North American Birds, Part 1. Ann Arbor, Michigan: Braum-Brumfield Inc; 1997.

[29] Stewart RM. Age and Crown types in the Golden-crowned Sparrow. West Bird Bander. 1972;47: 32–33.

[30] Griffiths R, Double MC, Orr K, Dawson R. A DNA test to sex most birds. Mol Ecol. 1998;7: 1071–1075. doi: 10.1046/j.1365-294x.1998.00389.x 9711866

[31] Chaine AS, Tjernell KA, Shizka D, Lyon BE. Sparrows use multiple status signals in winter social flocks. Anim Behav. 2011;81: 447–453.

[32] Lotek Wireless Inc. PinPoint Host user manual, rev. 10 PinPoint Host application for PinPoint GPS tags. 2018.

[33] Rappole JH, Tipton AR. New harness design for attachment of radio transmitters to small passerines. J F Ornithol. 1991;62: 335–337.

[34] Iverson AR, Humple DL, Cormier RL, Hull J. Land cover and NDVI are important predictors in habitat selection along migration for the Golden-crowned Sparrow, a temperate-zone migrating songbird. Mov Ecol. 2023;11: 1–19. doi: 10.1186/s40462-022-00353-2 36639697 PMC9837890

[35] Block TA, Lyon BE, Mikalonis Z, Chaine AS, Shizuka D. Social connections across migration: do Golden-crowned Sparrows that socialize in winter also breed together? Wilson J Ornithol. 2024;2024. Available: doi: 10.1676/23-00014

[36] Downs JA, Horner MW. Effects of Point Pattern Shape on Home‐Range Estimates. J Wildl Manage. 2008;72: 1813–1818. doi: 10.2193/2007-454

[37] Worton BJ. A review of models of home range for animal movement. Ecol Modell. 1987;38: 277–298.

[38] Worton BJ. Kernel methods for estimating the utilization distribution in home-range studies. Ecology. 1989;70: 164–168.

[39] Seaman DE, Millspaugh JJ, Kernohan BJ, Brundige GC, Raedeke KJ, Gitzen RA. Effects of Sample Size on Kernel Home Range Estimates. J Wildl Manage. 1999;63: 739. doi: 10.2307/3802664

[40] Hemson G, Johnson P, South A, Kenward R, Ripley R, Mcdonald D. Are kernels the mustard? Data from global positioning system (GPS) collars suggests problems for kernel home-range analyses with least-squares cross-validation. J Anim Ecol. 2005;74: 455–463. doi: 10.1111/j.1365-2656.2005.00944.x

[41] Gitzen RA, Millspaugh JJ, Kernohan BJ. Bandwidth Selection for Fixed-Kernel Analysis of Animal Utilization Distributions. J Wildl Manage. 2006;70: 1334–1344.

[42] Börger L, Franconi N, De Michele G, Gantz A, Meschi F, Manica A, et al. Effects of sampling regime on the mean and variance of home range size estimates. J Anim Ecol. 2006;75: 1393–1405. doi: 10.1111/j.1365-2656.2006.01164.x 17032372

[43] Niebuhr B, Mokross K, Silveira NS. movecology: mov ecology codes 1.0. San Francisco: Github; 2020.

[44] Calenge C. The package adehabitat for the R software: a tool for the analysis of space and habitat use by animals. Ecol Modell. 2006;197: 516–519.

[45] Havlíček J, Riegert J, Fuchs R. A comparison of foraging-range sizes, flight distances and foraging habitat preferences in urban and rural House Sparrow (Passer domesticus) populations. Ibis (Lond 1859). 2022;164: 1227–1242. doi: 10.1111/ibi.13072

[46] Seaman DE, Powell RA. An evaluation of the accuracy of kernel density estimators for home range analysis. Ecology. 1996;77: 2075–2085. doi: 10.2307/2265701

[47] Walcott J, Eckert S, Horrocks JA. Tracking hawksbill sea turtles (Eretmochelys imbricata) during inter-nesting intervals around Barbados. Mar Biol. 2012;159: 927–938. doi: 10.1007/s00227-011-1870-9

[48] ESRI. ArcGIS Desktop: Release 10. Redlands, CA: Environmental Systems Research Institute; 2011.

[49] Godet L, Harmange C, Marquet M, Joyeux E, Fournier J. Differences in home-range sizes of a bird species in its original, refuge and substitution habitats: challenges to conservation in anthropogenic habitats. Biodivers Conserv. 2018;27: 719–732. doi: 10.1007/s10531-017-1460-3

[50] Skinner AA, Matthews SN, Ward MP, Souza-Cole I, Wright JR, Thompson FR, et al. Eastern Whip-poor-wills have larger nonbreeding home ranges in areas with more agriculture and forest fragmentation. Ornithol Appl. 2023;125: 1–15. doi: 10.1093/ornithapp/duac050

[51] Natural Resources Canada (NRCan), Canada Centre for Remote Sensing (CCRS), Canada Centre for Mapping and EarthObservation (CCMEO), United States Geological Survey, Instituto Nacional de Estadística y Geografía (INEGI), Comisión Nacional para el Conocimiento y Uso de la Biodiversidad (CONABIO), et al. [NALCMS] 2020 North American land cover at 30 m spatial resolution. In: America Land Change Monitoring System Map [Internet]. 2023. Available: http://www.cec.org/north-american-environmental-atlas/land-cover-30m-2020/

[52] Hollister JW. elevatr: Access Elevation Data from Various APIs. R package version 0.4.1. 2021. Available: https://cran.r-project.org/package=elevatr/

[53] Riley SJ, DeGloria SD, Elliot R. A terrain ruggedness index that quantifies topographic heterogeneity. Intermountain Journal of Science. 1999. pp. 23–27.

[54] R Core Team. R: A language and environment for statistical computing. Vienna, Austria: R Foundation for Statistical Computing; 2021. Available: www.R-project.org

[55] Gotelli NJ, Ellison AM. A Primer of Ecological Statistics. Sinauer Associates; 2004.

[56] Lüdecke D, Ben-Shachar ME, Patil I, Waggoner P, Makowski D. performance: An R package for assessment, comparison and testing of statistical models. J Open Source Softw. 2021;6: 3139.

[57] Signer J, Fieberg J, Avgar T. Animal movement tools (amt): R package for managing tracking data and conducting habitat selection analyses. Ecol Evol. 2019;9: 880–890. doi: 10.1002/ece3.4823 30766677 PMC6362447

[58] Fieberg J, Kochanny CO. Quantifying Home-Range Overlap: the Importance of the Utilization Distribution. J Wildl Manage. 2005;69: 1346–1359. doi: 10.2193/0022-541x(2005)69[1346:qhotio]2.0.co;2

[59] Winner K, Noonan MJ, Fleming CH, Olson KA, Mueller T, Sheldon D, et al. Statistical inference for home range overlap. Methods Ecol Evol. 2018;9: 1679–1691. doi: 10.1111/2041-210X.13027

[60] Block TA. Demographic and social influence on the winter ecology of a migratory songbird, the Golden-crowned Sparrow (Zonotrichia atricapilla). University of California Santa Cruz. 2021.

[61] Iverson AR, Humple DL, Cormier RL, Hahn TP, Hull EM. Data from: Winter GPS tagging reveals home ranges on during the breeding season for a boreal-nesting migrant songbird, the Golden-crowned Sparrow. In: Movebank Data Repository. 2024. 10.1371/journal.pone.0305369 PMC11168665 38865434

[62] Block TA, Lyon BE, Mikalonis Z, Chaine AS, Shizuka D. Social connections across migration: Do Golden-crowned sparrows (Zonotrichia atricapilla) that socialize in winter also breed together? [Dataset]. In: Dryad [Internet]. 2023. Available: 10.7291/D1R08G

[63] Ralph C. J, Pearson CA. Correlation of Age, Size of Territory, Plumage, and Breeding Success in White-Crowned Sparrows. Condor. 1971;73: 77–80.

[64] Patterson TL, Petrinovich L. Territory Size in the White-Crowned Sparrow (Zonotrichia leucophrys): Measurement and Stability. Condor. 1978;80: 97–98. doi: 10.2307/1367796

[65] Formica VA., Gonser RA., Ramsay S, Tuttle EM. Spatial Dynamics of Alternative Reproductive Strategies: The Role of Neighbors. Ecology. 2004;85: 1125–1136.

[66] Rousseau P, Desrochers A, Hadley AS. Habitat selection and fidelity by white-throated sparrows (Zonotrichia albicollis): Generalist species, specialist individuals? Can J Zool. 2012;90: 595–601. doi: 10.1139/Z2012-025

[67] Sagario MC, Cueto VR. Seasonal space use and territory size of resident sparrows in the central monte desert, Argentina. Ardeola. 2014;61: 153–159. doi: 10.13157/arla.61.1.2014.153

[68] Sanders JL. Nest and roost site selection of Mountain White-crowned Sparrows (Zonotrichia leucophrys oriantha) in the Central Sierra Nevada. Princeton University. 1998.

[69] Morton ML. Late-season Events. The Mountain White-crowned Sparrow: Migation and reproduction at high altitude. Lawrence, Kansas: Allen Press Inc; 2002. pp. 179–204.

[70] Gow EA, Stutchbury BJM. Within-season nesting dispersal and molt dispersal are linked to habitat shifts in a Neotropical migratory songbird. Wilson J Ornithol. 2013;125: 696–708. doi: 10.1676/13-015.1

[71] Tonra CM, Wright JR, Matthews SN. Remote estimation of overwintering home ranges in an elusive, migratory nocturnal bird. Ecol Evol. 2019;9: 12586–12599. doi: 10.1002/ece3.5723 31788199 PMC6875585

[72] Monterroso P, Sillero N, Rosalino LM, Loureiro F, Alves PC. Estimating home-range size: When to include a third dimension? Ecol Evol. 2013;3: 2285–2295. doi: 10.1002/ece3.590 23919170 PMC3728965

[73] Miller TA, Brooks RP, Lanzone MJ, Cooper J, O’Malley K, Brandes D, et al. Summer and winter space use and home range characteristics of Golden Eagles (Aquila chrysaetos) in eastern North America. Condor. 2017;119: 697–719. doi: 10.1650/CONDOR-16-154.1

[74] Penteriani V, Ferrer M, Delgado MM. Floater strategies and dynamics in birds, and their importance in conservation biology: towards an understanding of nonbreeders in avian populations. Anim Conserv. 2011;14: 233–241. doi: 10.1111/j.1469-1795.2010.00433.x

[75] Hendricks P. Breeding biology and nestling development of Golden-crowned Sparrows in Alaska. Wilson Bull. 1987;99: 696–699.

[76] Swarth HS. Report on a collection of birds and mammals from the Atlin region, northern British Columbia. Berkeley; 1926.

[77] Kokko H, Gunnarsson TG, Morrell LJ, Gill JA. Why do female migratory birds arrive later than males? J Anim Ecol. 2006;75: 1293–1303. doi: 10.1111/j.1365-2656.2006.01151.x 17032361

[78] Van Horne B. Density as a Misleading Indicator of Habitat Quality. J Wildl Manage. 1983;47: 893–901.

[79] Looff HB. Returns of Golden-Crowned and Kadiak Fox Sparrows to Breeding Grounds on Kodiak Island, Alaska. Bird-Banding. 1939;10: 85–88.

[80] Seavy NE, Humple DL, Cormier RL, Gardali T. Establishing the breeding provenance of a temperate-wintering north american passerine, the golden-crowned sparrow, using light-level geolocation. PLoS One. 2012;7: 1–6. doi: 10.1371/journal.pone.0034886 22506055 PMC3323592

[81] Cormier RL, Humple DL, Gardali T, Seavy NE. Migratory connectivity of Golden-crowned Sparrows from two wintering regions in California. Anim Migr. 2016;3: 48–56. doi: 10.1515/ami-2016-0005

[82] Shizuka D, Chaine AS, Anderson J, Johnson O, Laursen IM, Lyon BE. Across-year social stability shapes network structure in wintering migrant sparrows. Ecol Lett. 2014;17: 998–1007. doi: 10.1111/ele.12304 24894316

[83] Madsen AE, Lyon BE, Chaine AS, Block TA, Shizuka D. Loss of flockmates weakens winter site fidelity in golden-crowned sparrows (Zonotrichia atricapilla). Proc Natl Acad Sci. 2023;120: e219939120. doi: 10.1073/pnas.2219939120 37523568 PMC10410770

[84] Alerstam T. Reoriented Bird Migration in Coastal Areas: Dispersal to Suitable Resting Grounds? Oikos. 1978;30: 405–408.

[85] Richardson WJ. Northeastward reverse migration of birds over Nova Scotia, Canada, in autumn—A radar study. Behav Ecol Sociobiol. 1982;10: 193–206. doi: 10.1007/BF00299685

[86] Nilsson C, Sjöberg S. Causes and characteristics of reverse bird migration: An analysis based on radar, radio tracking and ringing at Falsterbo, Sweden. J Avian Biol. 2016;47: 354–362. doi: 10.1111/jav.00707

[87] Crysler ZJ, Ronconi RA, Taylor PD. Differential fall migratory routes of adult and juvenile Ipswich Sparrows (Passerculus sandwichensis princeps). Mov Ecol. 2016;4: 1–8. doi: 10.1186/s40462-016-0067-8 26819707 PMC4729120

[88] Åkesson S, Morin J, Muheim R, Ottosson U. Dramatic orientation shift of white-crowned sparrows displaced across longitudes in the high arctic. Curr Biol. 2005;15: 1591–1597. doi: 10.1016/j.cub.2005.07.027 16139216

[89] Deutschlander ME, Muheim R. Fuel reserves affect migratory orientation of thrushes and sparrows both before and after crossing an ecological barrier near their breeding grounds. J Avian Biol. 2009;40: 85–89. doi: 10.1111/j.1600-048X.2008.04343.x

[90] Barve S, Hagemeyer ND, Winter RE, Chamberlain SD, Koenig WD, Winkler D., et al. Wandering woodpeckers: foray behavior in a social bird. Ecology. 2020;101: e02943. doi: 10.1002/ecy.2943 31782526

[91] Hudson EJ, Hahn M, Shizuka D. Nestling and adult sparrows respond differently to conspecific dialects. Behav Ecol. 2019;30: 48–56.

