# Supplementary material for: Winter GPS tagging reveals home ranges during the breeding season for a boreal-nesting migrant songbird, the Golden-crowned Sparrow
Source: PLoS One. 2024 Jun 12;19(6):e0305369. doi: 10.1371/journal.pone.0305369 (PMC11168665; doi:10.1371/journal.pone.0305369)
Supplement: S1 Fig — Each set of GPS points for each bird (with at least 10 points) was randomly sampled 20 times at various sample sizes (from five to the total points available), and KDEs were created at each sample size. From this, we decided on a cutoff of 13 points for creating home range estimates. Each curve title refers to the tag number. (PDF) [file pone.0305369.s001.pdf]

S1 Fig. Accumulation curves for Golden-crowned Sparrow breeding locations and home range size (50% KDEs and 95% KDEs), for Golden-crowned Sparrows (*Zonotrichia atricapilla)* GPS-tagged at wintering grounds in California 2017-2020. Each set of GPS points for each bird (with at least 10 points) was randomly sampled 20 times at various sample sizes (from five to the total points available), and KDEs were created at each sample size. From this, we decided on a cutoff of 13 points for creating home range estimates. Each curve title refers to the tag number.

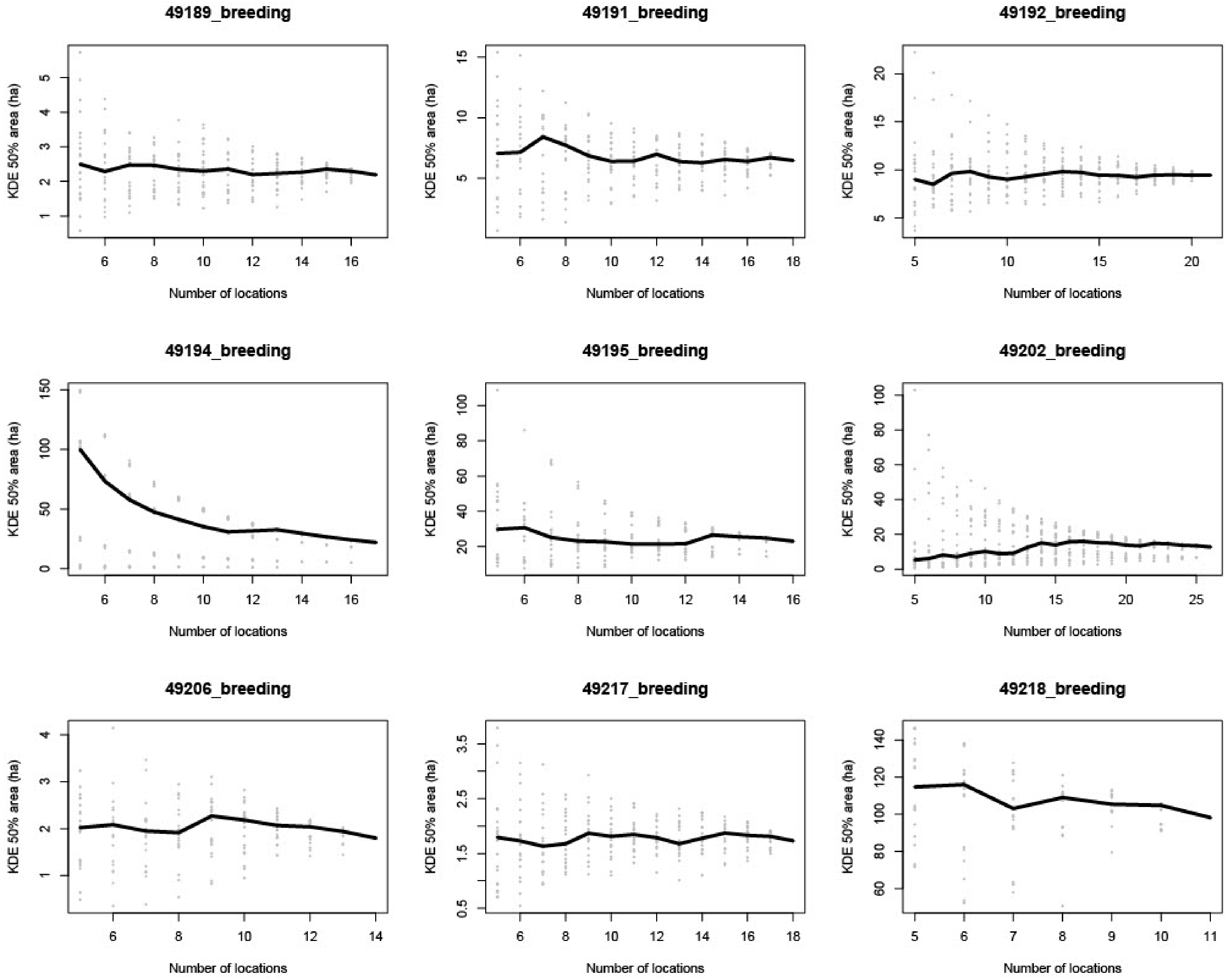
49189_breeding
49191_breeding
49192_breeding
49194_breeding
49195_breeding
49202_breeding
49206_breeding
49217_breeding
49218_breeding
KDE 50% area (ha)
Number of locations

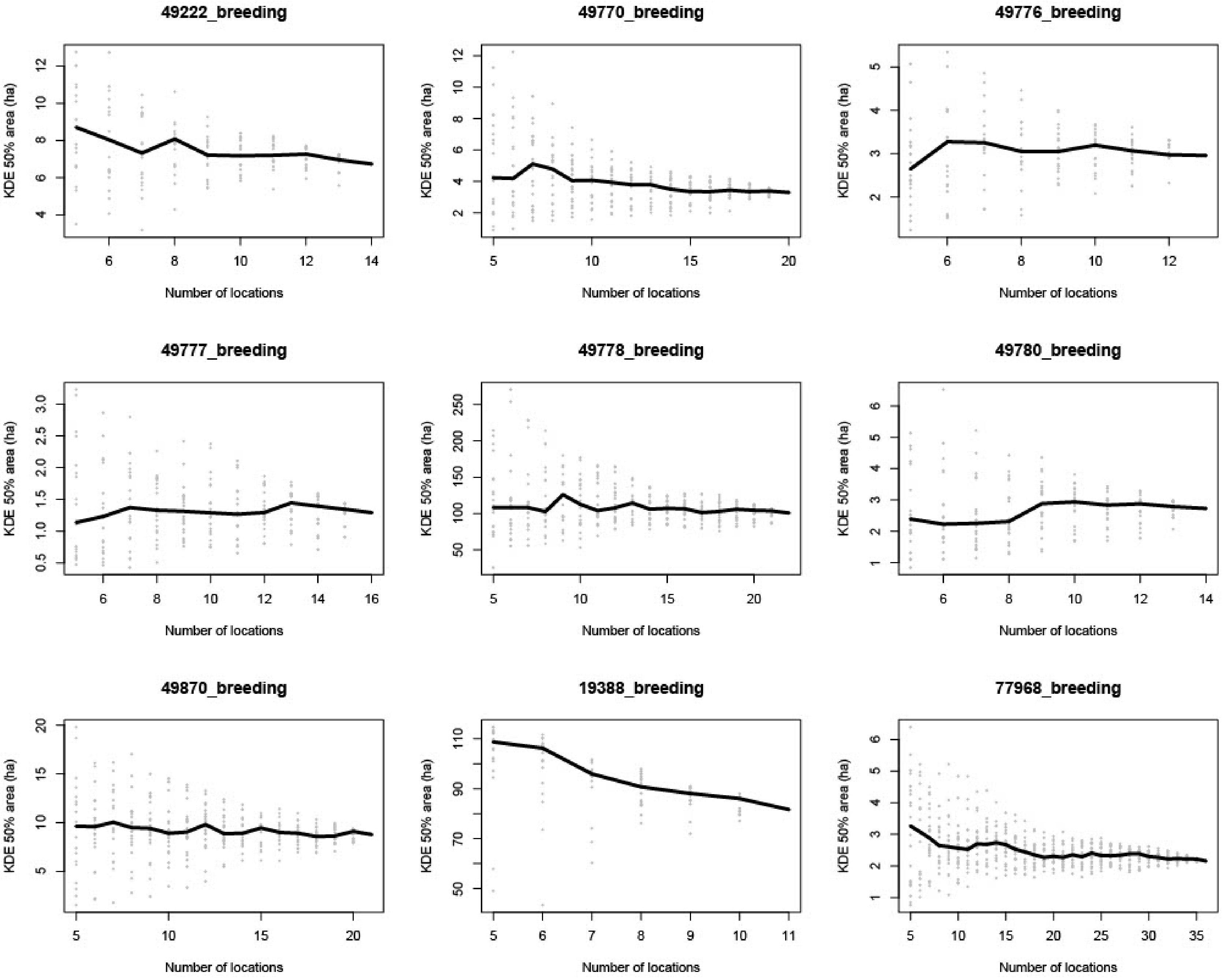
49222_breeding
KDE 50% area (ha)
4 6 8 10 12
6 8 10 12 14
Number of locations
49770_breeding
KDE 50% area (ha)
2 4 6 8 10 12
5 10 15 20
Number of locations
49776_breeding
KDE 50% area (ha)
2 3 4 5
6 8 10 12
Number of locations
49777_breeding
KDE 50% area (ha)
0.5 1.0 1.5 2.0 2.5 3.0
6 8 10 12 14 16
Number of locations
49778_breeding
KDE 50% area (ha)
50 100 150 200 250
5 10 15 20
Number of locations
49780_breeding
KDE 50% area (ha)
1 2 3 4 5 6
6 8 10 12 14
Number of locations
49870_breeding
KDE 50% area (ha)
5 10 15 20
5 10 15 20
Number of locations
19388_breeding
KDE 50% area (ha)
50 70 90 110
5 6 7 8 9 10 11
Number of locations
77968_breeding
KDE 50% area (ha)
1 2 3 4 5 6
5 10 15 20 25 30 35
Number of locations

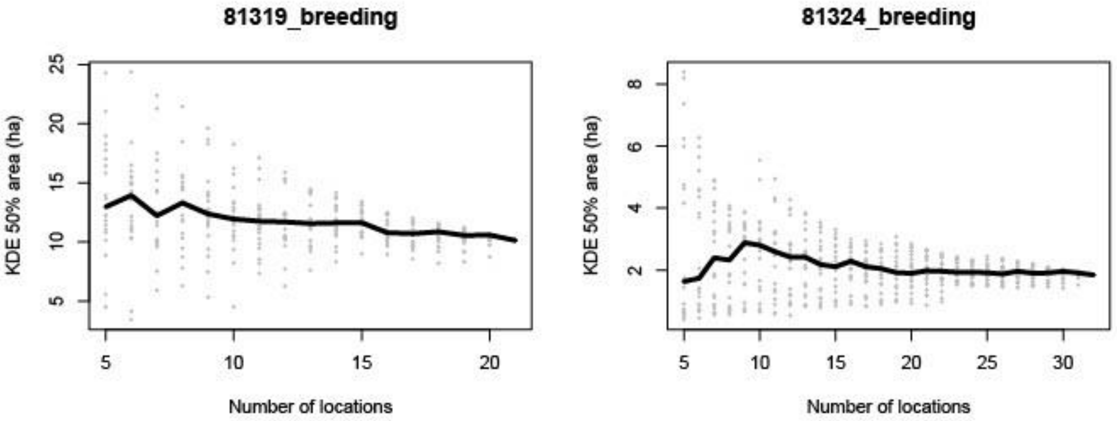

81319_breeding
KDE 50% area (ha)
Number of locations
81324_breeding
KDE 50% area (ha)
Number of locations

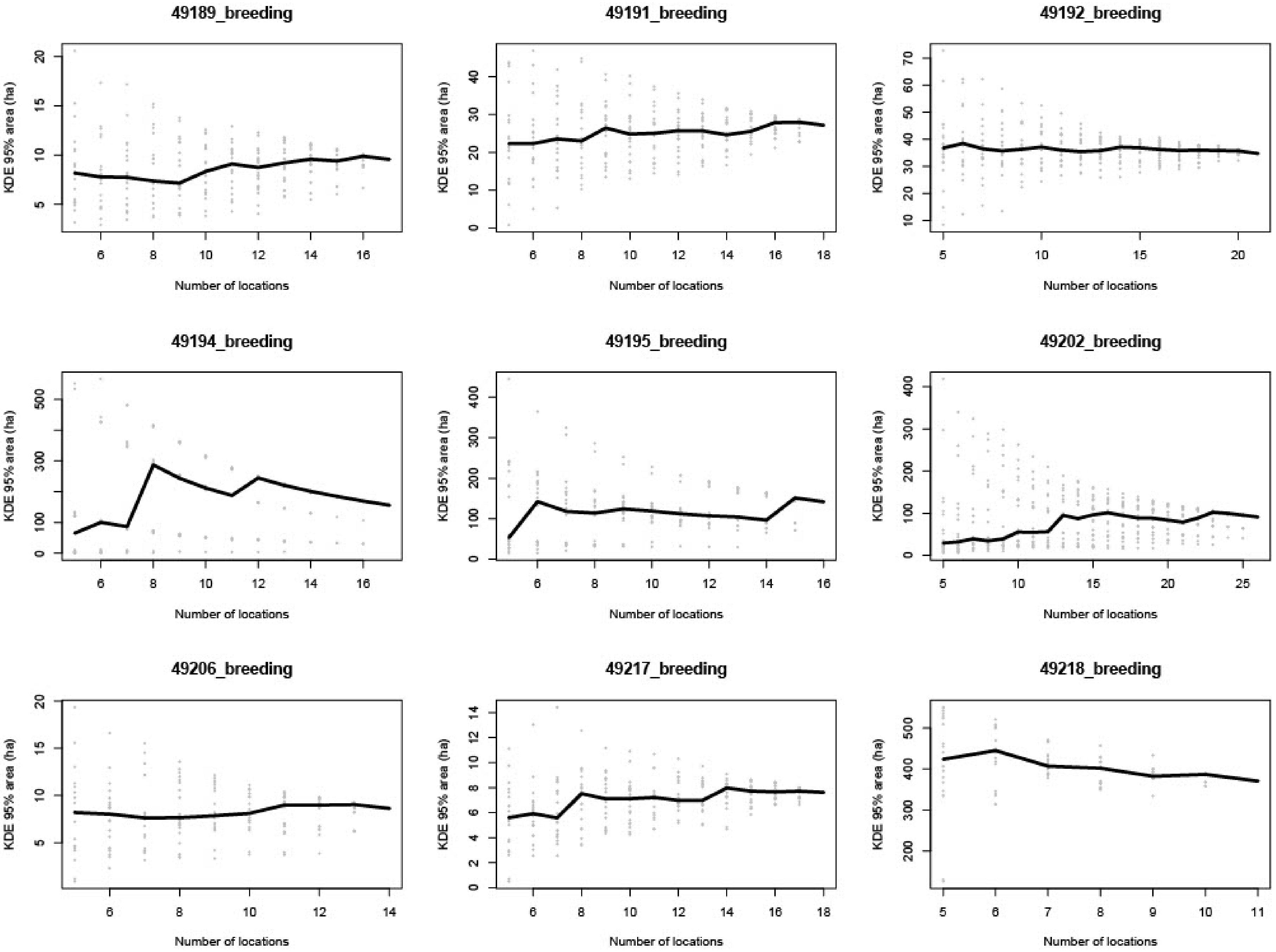
49189_breeding
49191_breeding
49192_breeding
49194_breeding
49195_breeding
49202_breeding
49206_breeding
49217_breeding
49218_breeding
KDE 95% area (ha)
Number of locations

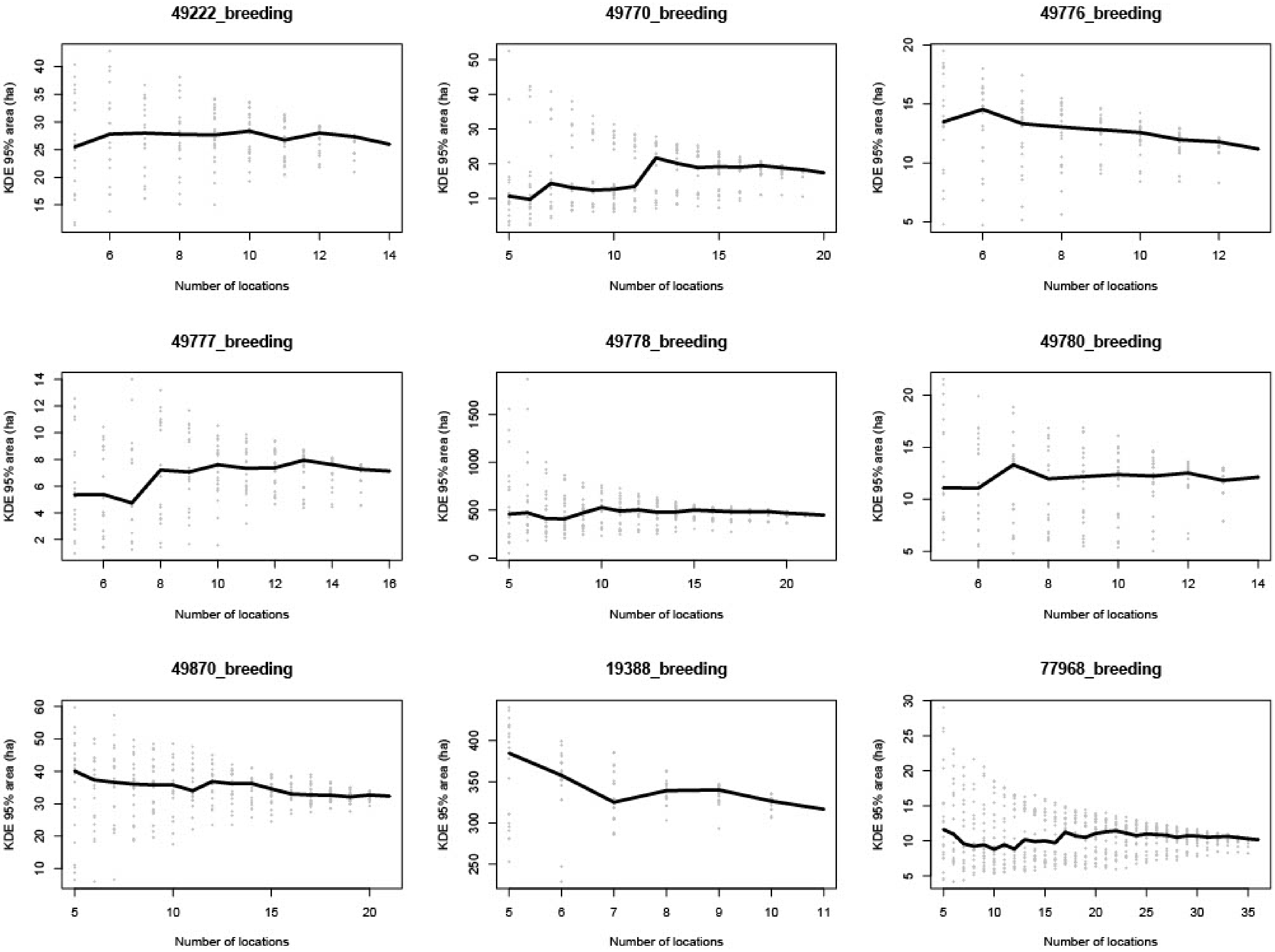
49222_breeding
KDE 95% area (ha)
15 20 25 30 35 40
6 8 10 12 14
Number of locations
49770_breeding
KDE 95% area (ha)
10 20 30 40 50
5 10 15 20
Number of locations
49776_breeding
KDE 95% area (ha)
5 10 15 20
6 8 10 12
Number of locations
49777_breeding
KDE 95% area (ha)
2 4 6 8 10 12 14
6 8 10 12 14 16
Number of locations
49778_breeding
KDE 95% area (ha)
0 500 1000 1500
5 10 15 20
Number of locations
49780_breeding
KDE 95% area (ha)
5 10 15 20
6 8 10 12 14
Number of locations
49870_breeding
KDE 95% area (ha)
10 20 30 40 50 60
5 10 15 20
Number of locations
19388_breeding
KDE 95% area (ha)
250 300 350 400
5 6 7 8 9 10 11
Number of locations
77968_breeding
KDE 95% area (ha)
5 10 15 20 25 30
5 10 15 20 25 30 35
Number of locations

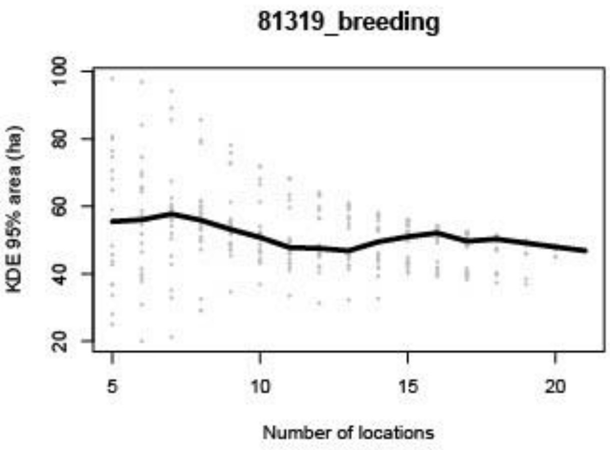
81319_breeding
KDE 95% area (ha)
Number of locations

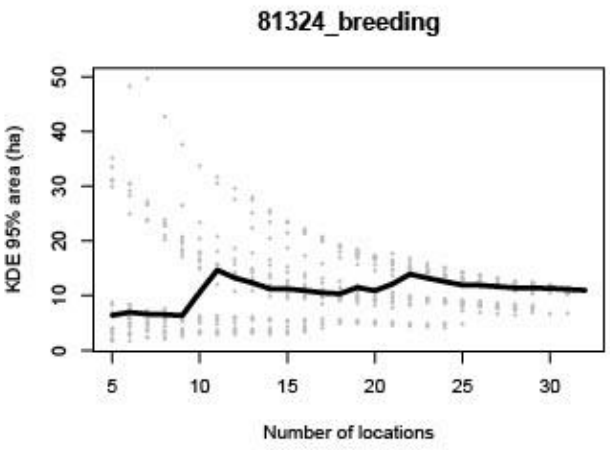
81324_breeding
KDE 95% area (ha)
Number of locations
