# Supplementary material for: Winter GPS tagging reveals home ranges during the breeding season for a boreal-nesting migrant songbird, the Golden-crowned Sparrow
Source: PLoS One. 2024 Jun 12;19(6):e0305369. doi: 10.1371/journal.pone.0305369 (PMC11168665; doi:10.1371/journal.pone.0305369)
Supplement: S3 Fig — Throughout the breeding area of Golden-crowned Sparrows GPS-tagged at wintering grounds in California 2017–2020, shrubland was widely available. This figure shows a close-up of breeding centroids (pink circles) in Alaska, and shrubland areas are shown in brown tones (land cover classes 7, 8, and 11). The full land cover names for this layer are as follows (not all possible types are shown in this map): 1 = Temperate or sub-polar needleleaf forest; 2 = Sub-polar taiga needleleaf forest; 3 = Tropical or sub-tropical broadleaf evergreen forest; 4 = Tropical or sub-tropical broadleaf deciduous forest; 5 = Tropical or sub-polar broadleaf deciduous forest; 6 = Mixed forest; 7 = Tropical or sub-tropical shrubland; 8 = Temperate or sub-polar shrubland; 9 = Tropical or sub-tropical grassland; 10 = Temperate or sub-polar grassland; 11 = Sub-polar or polar shrubland-lichen-moss; 12 = Sub-polar or polar grassland-lichen-moss; 13 = Sub-polar or polar barren-lichen-moss; 14 = Wetland; 15 = Cropland; 16 = Barren land; 17 = Urban and built up; 18 = Water; 19 = Snow and ice. (PDF) [file pone.0305369.s003.pdf]

S3 Fig. Land cover classes in Alaska.

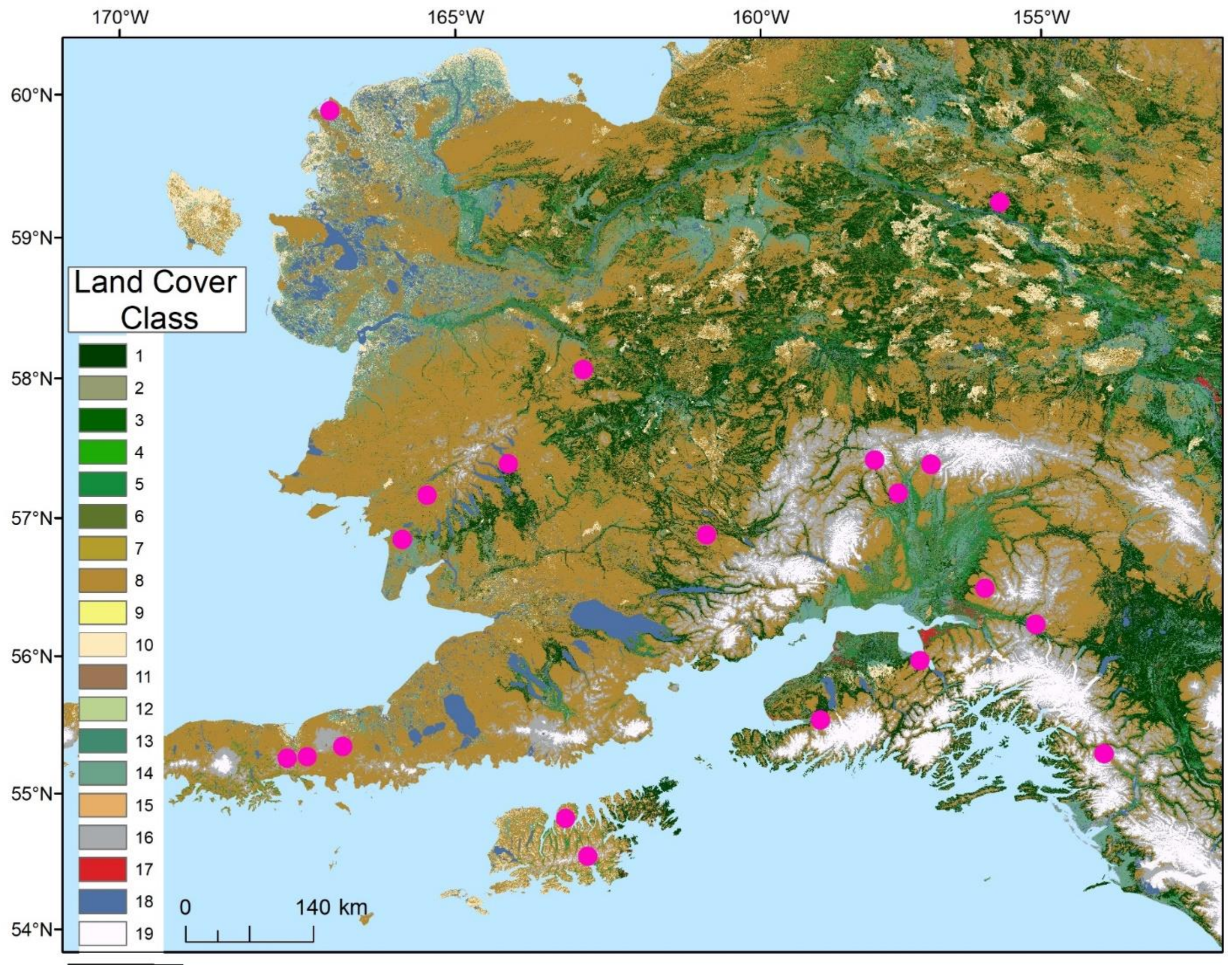

Throughout the breeding area of Golden-crowned Sparrows GPS-tagged at wintering grounds in California 2017-2020, shrubland was widely available. This figure shows a close-up of breeding centroids (pink circles) in Alaska, and shrubland areas are shown in brown tones (land cover classes 7, 8, and 11). The full land cover names for this layer are as follows (not all possible types are shown in this map): 1 = Temperate or sub-polar needleleaf forest; 2 = Sub-polar taiga needleleaf forest; 3 = Tropical or sub-tropical broadleaf evergreen forest; 4 = Tropical or sub-tropical broadleaf deciduous forest; 5 = Tropical or sub-polar broadleaf deciduous forest; 6 = Mixed forest; 7 = Tropical or sub-tropical shrubland; 8 = Temperate or sub-polar shrubland; 9 = Tropical or sub-tropical grassland; 10 = Temperate or sub-polar grassland; 11 = Sub-polar or polar shrubland-lichen-moss; 12 = Sub-polar or polar grassland-lichen-moss; 13 = Sub-polar or polar barren-lichen-moss; 14 = Wetland; 15 = Cropland; 16 = Barren land; 17 = Urban and built up; 18 = Water; 19 = Snow and ice. Land cover classes are from the 2020 North America Land Change Monitoring System map (NALCMS 2023).

Natural Resources Canada (NRCan), Canada Centre for Remote Sensing (CCRS), Canada Centre for Mapping and EarthObservation (CCMEO), United States Geological Survey, Instituto

Nacional de Estadística y Geografía (INEGI), Comisión Nacional para el Conocimiento y Uso de la Biodiversidad (CONABIO), et al.[NALCMS] 2020 North American land cover at 30 m spatial resolution. In: America Land Change Monitoring System Map [Internet]. 2023. Available: http://www.cec.org/north-american-environmental-atlas/land-cover-30m-2020/
