# Supplementary material for: Winter GPS tagging reveals home ranges during the breeding season for a boreal-nesting migrant songbird, the Golden-crowned Sparrow
Source: PLoS One. 2024 Jun 12;19(6):e0305369. doi: 10.1371/journal.pone.0305369 (PMC11168665; doi:10.1371/journal.pone.0305369)
Supplement: S4 Fig — Five birds showed reverse migration, flying in the opposite direction expected for migration (i.e. south or west). Yellow dots and polygons represent final destinations (centroids, and home ranges where visible). Green points are migration locations and are connected by a purple line to demonstrate directionality for the birds. (PDF) [file pone.0305369.s004.pdf]

S4 Fig. Reverse migration for Golden-crowned Sparrows (*Zonotrichia atricapilla*) GPS-tagged at wintering grounds in California 2017-2020.

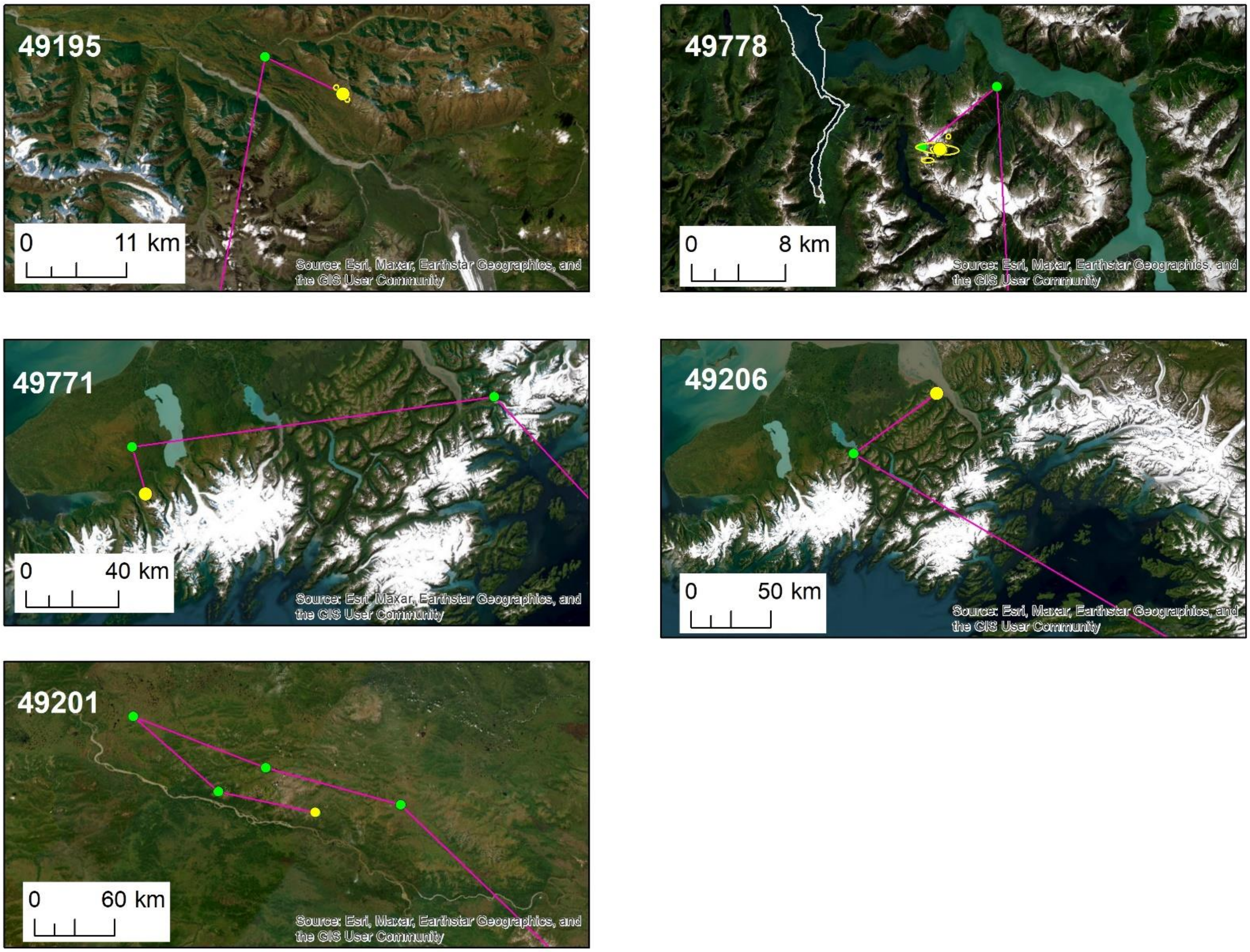

Five birds showed reverse migration, flying in the opposite direction expected for migration (i.e. south or west). Yellow dots and polygons represent final destinations (centroids, and home ranges where visible). Green points are migration locations and are connected by a purple line to demonstrate directionality for the birds.
