# Supplementary material for: Winter GPS tagging reveals home ranges during the breeding season for a boreal-nesting migrant songbird, the Golden-crowned Sparrow
Source: PLoS One. 2024 Jun 12;19(6):e0305369. doi: 10.1371/journal.pone.0305369 (PMC11168665; doi:10.1371/journal.pone.0305369)

S5 Fig. The distance to centroids (created from kernel density estimates [KDE]) from each location (date given on x axis for each location) on the breeding grounds for Golden-crowned Sparrows (*Zonotrichia atricapilla*) GPS-tagged at wintering grounds in California from 2017-2020. This figure shows 355 points across 19 KDEs (see Table 1 for details on all KDEs).

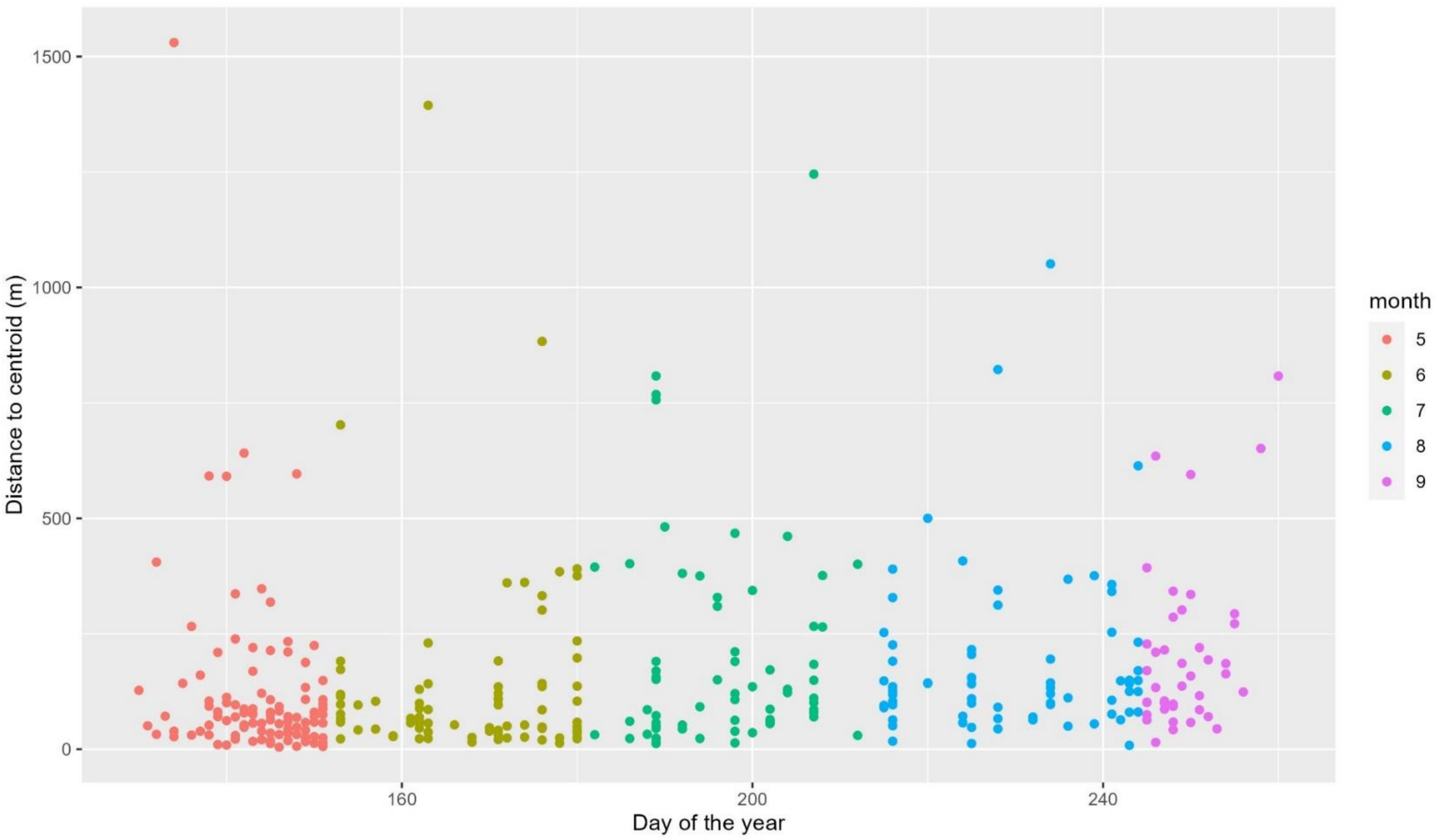

Supplement: S5 Fig — This figure shows 355 points across 19 KDEs (see Table 1 for details on all KDEs). (PDF) [file pone.0305369.s005.pdf]
