# Supplementary material for: Winter GPS tagging reveals home ranges during the breeding season for a boreal-nesting migrant songbird, the Golden-crowned Sparrow
Source: PLoS One. 2024 Jun 12;19(6):e0305369. doi: 10.1371/journal.pone.0305369 (PMC11168665; doi:10.1371/journal.pone.0305369)
Supplement: S1 Table — For two tags, there were two core areas and a center point is given for each. (PDF) [file pone.0305369.s006.pdf]

S1 Table. Home range center points (centroids of 50% KDEs) for Golden-crowned Sparrows (*Zonotrichia atricapilla*) GPS-tagged at wintering grounds in California 2017-2020.

Tag Number	Longitude	Latitude
49189	-145.576	61.078
49191	-152.970	57.540
49192	-151.919	62.201
49194	-159.346	58.943
49195	-148.100	61.836
49202	-128.344	53.388
49206	-149.702	60.919
49217	-157.843	57.008
49222	-159.500	59.431
49770	-148.103	61.836
49776	-145.576	61.078
49777	-158.519	56.595
49778	-128.346	53.387
49780	-158.602	61.358
49870 (1)	-151.688	62.624
49870 (2)	-151.694	62.623
77968	-158.247	56.727
81319 (1)	-152.704	62.345
81319 (2)	-152.710	62.344
81324	-153.714	57.717

For two tags, there were two core areas and a center point is given for each.
