# Supplementary material for: Winter GPS tagging reveals home ranges during the breeding season for a boreal-nesting migrant songbird, the Golden-crowned Sparrow
Source: PLoS One. 2024 Jun 12;19(6):e0305369. doi: 10.1371/journal.pone.0305369 (PMC11168665; doi:10.1371/journal.pone.0305369)
Supplement: S2 Table — Land cover classes are from the 2020 North America Land Change Monitoring System map (NALCMS 2023). Needleleaf forest = “Temperate or sub-polar needleleaf forest”; Broadleaf forest = “Temperate or sub-polar broadleaf deciduous forest”; Shrubland = “Temperate or sub-polar shrubland”; Grassland = “Temperate of sub-polar grassland”; Shrubland-lichen-moss = “Sub-polar or polar shrubland-lichen-moss”. (PDF) [file pone.0305369.s007.pdf]

S2 Table. Land cover classes (the number of cells 30m x 30m) represented in each home range (50% and 95% KDEs), along with the proportion of shrubland represented across all cells, for Golden-crowned Sparrows (*Zonotrichia atricapilla*) GPS-tagged at wintering grounds in California 2017-2020.

Tag	Bird	Needleleaf forest	Broadleaf forest	Shrubland	Grassland	Shrubland-lichen-moss	Barren lands	Water	Snow and Ice	Total Cells	Proportion Shrubland
**50% KDEs**											
49189	Q	0	0	25	0	0	0	0	0	25	1.0
49191	I	0	0	53	16	0	6	0	0	75	0.7
49192	J	0	0	103	0	0	0	0	0	103	1.0
49194	K	0	1	35	0	0	0	0	0	36	1.0
49195	R	3	0	233	0	2	17	0	0	255	0.9
49202	S	0	0	17	41	25	58	0	0	141	0.1
49206	F	0	0	21	0	0	0	0	0	21	1.0
49217	M	0	0	19	0	0	0	0	0	19	1.0
49222	C	7	11	58	0	0	0	0	0	76	0.8
49770	R	0	0	35	0	0	0	0	0	35	1.0
49776	Q	0	0	31	0	0	0	0	0	31	1.0
49777	N	0	6	8	0	0	0	0	0	14	0.6
49778	S	0	0	171	68	25	382	0	32	678	0.3
49780	O	11	0	15	0	0	0	0	0	26	0.6
49870	P	0	0	75	0	0	0	0	0	75	1.0
77968	U	0	0	24	0	0	0	0	0	24	1.0
81319	V	0	0	95	0	0	7	0	0	102	0.9
81324	W	0	0	21	0	0	0	0	0	21	1.0
**95% KDEs**											
49189	Q	0	0	104	0	0	0	0	0	104	1.0
49191	I	0	0	196	100	0	8	0	0	304	0.6
49192	J	0	0	386	0	0	0	0	0	386	1.0

49194	K	0	17	235	0	0	0	0	0	252	0.9
49195	R	7	0	1313	0	19	239	0	0	1578	0.8
49202	S	0	0	276	69	35	551	0	83	1014	0.3
49206	F	0	0	95	0	0	0	0	0	95	1.0
49217	M	0	0	82	0	0	0	0	0	82	1.0
49222	C	17	19	237	0	0	9	0	5	287	0.8
49770	R	7	0	181	0	2	2	0	0	192	0.9
49776	Q	0	0	120	0	0	3	0	0	123	1.0
49777	N	0	23	53	0	0	0	1	0	77	0.7
49778	S	121	5	428	149	30	1771	0	657	3161	0.1
49780	O	32	0	97	0	0	0	0	0	129	0.8
49870	P	0	0	292	3	0	0	0	0	295	1.0
77968	U	0	0	111	0	0	0	0	0	111	1.0
81319	V	0	0	410	0	0	88	0	0	498	0.8
81324	W	0	0	113	10	0	0	0	0	123	0.9

Land cover classes are from the 2020 North America Land Change Monitoring System map (NALCMS 2023). Needleleaf forest = “Temperate or sub-polar needleleaf forest”; Broadleaf forest = “Temperate or sub-polar broadleaf deciduous forest”; Shrubland = “Temperate or sub-polar shrubland”; Grassland = “Temperate of sub-polar grassland”; Shrubland-lichen-moss = “Sub-polar or polar shrubland-lichen-moss”.

Natural Resources Canada (NRCan), Canada Centre for Remote Sensing (CCRS), Canada Centre for Mapping and EarthObservation (CCMEO), United States Geological Survey, Instituto Nacional de Estadística y Geografía (INEGI), Comisión Nacional para el Conocimiento y Uso de la Biodiversidad (CONABIO), et al.[NALCMS] 2020 North American land cover at 30 m spatial resolution. In: America Land Change Monitoring System Map [Internet]. 2023. Available: http://www.cec.org/north-american-environmental-atlas/land-cover-30m-2020/
