# Supplementary material for: Winter GPS tagging reveals home ranges during the breeding season for a boreal-nesting migrant songbird, the Golden-crowned Sparrow
Source: PLoS One. 2024 Jun 12;19(6):e0305369. doi: 10.1371/journal.pone.0305369 (PMC11168665; doi:10.1371/journal.pone.0305369)
Supplement: S1 File — (PDF) [file pone.0305369.s008.pdf]

**S1 File. Supplemental Methods.**

**Winter GPS tagging reveals home ranges during the breeding season for a boreal-nesting migrant songbird, the Golden-crowned Sparrow**

Autumn R. Iverson, Diana L. Humple, Renée L. Cormier, Thomas P. Hahn, Theadora A. Block, Daizaburo Shizuka, Bruce E. Lyon, Alexis S. Chaine, Emily J. Hudson, Elisha M. Hull

Swift fixes: An evaluation of Swift fixes in forested conditions, which could obstruct GPS signals and lead to less accurate fixes, showed that 80% of fixes were within 30 m of the actual tag location (95% within 271 m [1]). While that reported fix success rate is high, we expected Golden-crowned Sparrows to stay in more open habitat during breeding and therefore have an even higher rate of fix accuracy.

GPS point filtering; For filtering GPS points, first we removed points with poor HDOP values (HDOP >20) if they had fewer than five satellites (n = 6 points total across all individuals). Next, for points with fair HDOP values (20 ≥ HDOP >10), we removed any points with a 2D fix (3 satellites, *n* = 3 points across all individuals). For each set of points (per tag) we calculated the maximum distance between the highest quality points (HDOP < 5 and satellites > 3) which averaged 680 m across all birds. Therefore, our third filtering step included removing points with fair HDOP values and fewer than 5 satellites that also exceeded this average distance (n = 1 point). Lastly, two birds had points during the breeding period that were filtered out due to large distances to other points. For one bird (tag 77968), we removed one point that we considered an error as it was >300 km away from the main cluster of points and would have required travel over the ocean (improbable during its breeding season). For another bird (tag 49870), we removed a GPS point with a valid HDOP value and a high number of satellites because it was 6 km away from the main breeding cluster and while it might have represented legitimate movement outside the home range, it caused non-convergence of the kernel density estimate.

Accumulation curves: Accumulation curves were created by iteratively estimating 50% KDE and 95% KDE sizes for different subsets of points from each tag. The point subsets ranged from five to the maximum points available for each tag. At each point level (five to the maximum), points were randomly selected 20 times for the creation of the KDEs (i.e., for each tag we created 20 KDEs with five points, with points randomly selected each time, then 20 KDEs with six points, etc).

1. Forrest SW, Recio MR, Seddon PJ. Moving wildlife tracking forward under forested conditions with the SWIFT GPS algorithm. Anim Biotelemetry. 2022;10: 1–11. doi:10.1186/s40317-022-00289-9

**R Code**

```
## Code for Iverson et al. 2024, Plos One


#### ACCUMULATION CURVES  ####
#adapted from
https://github.com/bniebuhr/movecology/blob/master/home_range_accumulation_curves
/home_range_accumulation_curves.R

# where all_data_sp is a Spatial Data Frames object with columns: BirdID, DATE, season,
SID
# where BirdID is the tag ID, and SID is a combination of tag ID and season (e.g., "breeding",
"migration")
# All filtered locations are included for animals to attempt MCPs on
#requiring packages move and adehabitatHR

iterations <- 20

### MCP 95%
### Parameters to control
MCP_percentage <- 95
cumHRmcp <- list() ## List with cumulative sample size for all individuals
for (i in 1:length(ids)) {  ## loop for individuals
  print(i)
  cumHRmcp[[i]] <- list()
  for(j in 1:iterations) { # Loop for iterations
    temp <- all_data_sp[which(all_data_sp$SID == ids[i]),]
    # Here we do not use the points as they are, but randomize their order
    temp <- SpatialPoints(coordinates(temp)[sample(length(temp)),],
CRS(proj4string(all_data_sp)))
    cumulative <- vector()
    for(k in 5:length(temp)){  ##loop for sample size from 5 locations to all locations
      cumulative[k] <- mcp.area(temp[1:k,], percent = MCP_percentage, plotit = F)
    }
    cumHRmcp[[i]][[j]] <- data.frame(hr = unlist(cumulative), ssize = 5:length(temp))
  }
}

names(cumHRmcp) <- ids
cumHRmcp
```

```
# Plotting
par(mfrow = c(3,3))
# Seeing cumulative MCP area plots
for(i in 1:length(ids)) {
  cum.df <- as.data.frame(cumHRmcp[[i]])
  cumHR.df.mult <- cum.df[,c(2,seq(1, ncol(cum.df), 2))]

  cumHR.df.mult2 <- data.frame(npoins = rep(cumHR.df.mult[,1], iterations), HR =
unlist(c(cumHR.df.mult[,2:ncol(cumHR.df.mult)])))
  plot(cumHR.df.mult2$npoins, cumHR.df.mult2$HR, cex=0.5, pch=16, col = 'grey',
main=ids[i],
      xlab = "Number of locations",ylab = paste0("MCP ", MCP_percentage, "% area (ha)"))
  points(unique(cumHR.df.mult2$npoins), apply(cumHR.df.mult[,2:ncol(cumHR.df.mult)],
MARGIN = 1, FUN = median), type="l", lwd=3, lty=1)

  Sys.sleep(1)
}


## then for KDEs, 95% here as example
# where all_data_sp_kde is a Spatial Data Frames object with columns: BirdID,  DATE, type,
season, SID
# where BirdID is the tag ID, and SID is a combination of tag ID and season (e.g., "breeding",
"migration")
# All filtered locations are included for animals to attempt KDEs on

iterations <- 20


### Parameters to control
KDE_percentage <- 95
cumHRkde <- list() ## List with cumulative sample size for all individuals
for (i in 1:length(ids_k)) {  ## loop for individuals
  print(i)
  cumHRkde[[i]] <- list()
  for(j in 1:iterations) { # Loop for iterations
    temp <- all_data_sp_kde[which(all_data_sp_kde$SID == ids_k[i]),]
    # Here we do not use the points as they are, but randomize their order
    temp <- SpatialPoints(coordinates(temp)[sample(length(temp)),],
CRS(proj4string(all_data_sp_kde)))
    cumulative <- vector()
    for(k in 5:length(temp)){  ##loop for sample size from 5 locations to all locations
      UD <- kernelUD(temp[1:k,], h = "href", grid = 500, extent = 5)
```

```
      cumulative[k] <- kernel.area(UD, percent = KDE_percentage)
    }
    cumulative <- cumulative[5:length(cumulative)]
    cumHRkde[[i]][[j]] <- data.frame(hr = unlist(cumulative), ssize = 5:length(temp))
  }
}

names(cumHRkde) <- ids_k
cumHRkde


# Plotting
par(mfrow = c(3,3))
# Seeing cummulative kde area plots
for(i in 1:length(ids_k)) {
  cum.df <- as.data.frame(cumHRkde[[i]])
  cumHR.df.mult <- cum.df[,c(2,seq(1, ncol(cum.df), 2))]

  cumHR.df.mult2 <- data.frame(npoins = rep(cumHR.df.mult[,1], iterations), HR =
unlist(c(cumHR.df.mult[,2:ncol(cumHR.df.mult)])))
  plot(cumHR.df.mult2$npoins, cumHR.df.mult2$HR, cex=0.5, pch=16, col = 'grey',
main=ids_k[i],
       xlab = "Number of locations",ylab = paste0("KDE ", KDE_percentage, "% area (ha)"))
  points(unique(cumHR.df.mult2$npoins), apply(cumHR.df.mult[,2:ncol(cumHR.df.mult)],
MARGIN = 1, FUN = median), type="l", lwd=3, lty=1)

  Sys.sleep(1)
}




#### MCP/ KDEs ####

library(adehabitatHR)
library(maptools)
library(raster)
library(rgeos)
library(rgdal)


#######To run this code, you need to have a data file (in csv format) with the following
columns (using the exact names below (all uppercase)):
## ID - unique animal ID
```

```
## TID - unique track ID (to differentiate between multiple tracks for the same animal (for example, you can use the start date of the unique track as the TID))
## DATE - day of year in mm/dd/yyyy format
## POINT_X - x spatial coordinate
## POINT_Y - y spatial coordinate
####

#in the following example, "all_data" is the dataframe we are working with, and has these columns

# standard deviation ratio settings for rescaling KDEs
# if one dimension (x/y) is skewed more than [sd.ratio] times the other, KDEs will be rescaled
# minimum value is 1 (rescale all KDEs)
sd.ratio <- 1.5 #######Can optionally change this as needed

#MCP percentage (0 - 100)
mcp.per<-95            #######can optionally change this as needed

#KDE percentages
kde.per<-c(25,50,95) #######can optionally change this as needed


dir.create("utilization_distributions")  #change directory as needed

# set min and max sd.ratio for KDE rescaling
sd.ratio.min<-1/sd.ratio
sd.ratio.max<-1*sd.ratio

head<-c("unique_id","num_total_locs","num_meandailylocs","ratio_xy","href")
capture.output(head,file="utilization_distributions/ud_output.txt",append=T)

ctr<-0
list.ani<-unique(all_data$ID)
print(list.ani)

for (idnum in list.ani){

  #subset data for unique animal
  data<-subset(all_data,all_data$ID==as.character(idnum))

  for (u in unique(data$TID)){

    ctr<-ctr+1
```

```
  #subset data for unique animal track
  ani<-subset(data,data$TID==u)
  uniqid<-paste0(idnum,"_",gsub("/","_",u))
  print(uniqid)

  #calculate mean daily locations (if there is more than one point a day)
  mdl<-aggregate(cbind(ani$POINT_X,ani$POINT_Y),by=list(ani$DATE),FUN="mean")
  mdl.out<-cbind(uniqid,mdl)
  names(mdl.out)<-c("uniq_ID","date","point_x","point_y")

  # create output SPDF/text file for points, write points to text file
  if (ctr == 1) {
   write.table(mdl.out,file="mean_daily_locs_all.txt",append=F,col.names=T,row.names=F)
   mdl.shp <- SpatialPointsDataFrame(data.frame(x=mdl.out$point_x,y=mdl.out$point_y),
data = mdl.out)
  } else {
   write.table(mdl.out,file="mean_daily_locs_all.txt",append=T,col.names=F,row.names=F)
   mdl.shp <- rbind(mdl.shp,
SpatialPointsDataFrame(data.frame(x=mdl.out$point_x,y=mdl.out$point_y), data =
mdl.out))
  }

  print(paste0(length(mdl[,1])," mean daily locations."))

  #make spatialpoints with mean daily locations, and all points
  locs<-SpatialPoints(data.frame(x=mdl$V1,y=mdl$V2))
  alllocs<-SpatialPoints(data.frame(x=ani$POINT_X,y=ani$POINT_Y))

  if (length(alllocs$x)>4){  #input the minimum points for MCPs here

   #MCP (uses all locations)
   cp<-mcp(alllocs,percent=mcp.per)
   cp$id<-uniqid
   cp$percent <- mcp.per
   cp$type <- "MCP"
   writeOGR(cp,  dsn="utilization_distributions", layer = paste0(uniqid,"_mcp"), driver =
"ESRI Shapefile")

   if(length(locs$x)>12){  #input the minimum points for KDEs here
    #KDE (uses mean daily locations)
    #calculate x/y standard deviation ratio for KDE
    sd.x<-sd(locs$x)
    sd.y<-sd(locs$y)
    rat.xy<-sd.x/sd.y
```

```
	print(rat.xy)

	#if ratio is within specified limits, use regular locations, otherwise divide by standard
deviation
	if (rat.xy > sd.ratio.min & rat.xy < sd.ratio.max)  {locs.kde<-locs
	print("Using regular coordinates")

	#KDE *what grid number to choose (grid ?) *this is number of cells for longest
direction*
	kde<-kernelUD(locs.kde,h = "href",grid=100)
	h.val<-
c(uniqid,length(alllocs$x),length(locs$x),round(rat.xy,3),kde@h$convergence,round(kde@
h$h,4))
	print(h.val)
	vud <- getvolumeUD(kde)

	#get kernel density volume
	fud <- vud[[1]]
	hr<-as.data.frame(fud)[,1]

	hr2<-data.frame(hr)
	ka<-data.frame(x=(coordinates(vud)[,1]),y=(coordinates(vud)[,2]),z=hr2)
	kb<-rasterFromXYZ(ka,digits=8)
	kc<-rasterToContour(kb,maxpixels=3000000,levels=kde.per)
	kc$id<-uniqid

	#writeOGR(kc,  dsn="utilization_distributions", layer = paste0(uniqid,"_kde"), driver =
"ESRI Shapefile")
	#for KDEline output, uncomment above
	capture.output(h.val,file="utilization_distributions/ud_output.txt",append=T)}

	else {locs.kde<-SpatialPoints(data.frame(x=locs$x/sd.x,y=locs$y/sd.y))
	print("Using transformed coordinates")

	kde<-kernelUD(locs.kde,h = "href",grid=100)
	h.val<-
c(uniqid,length(alllocs$x),length(locs$x),round(rat.xy,3),kde@h$convergence,round(kde@
h$h,4))
	print(h.val)
	vud <- getvolumeUD(kde)

	#get kernel density volume
	fud <- vud[[1]]
	hr<-as.data.frame(fud)[,1]
```

```
    hr2<-data.frame(hr)
    ka<-data.frame(x=(coordinates(vud)[,1])*sd.x,y=(coordinates(vud)[,2])*sd.y,z=hr2)
    kb<-rasterFromXYZ(ka,digits=8)
    kc<-rasterToContour(kb,maxpixels=3000000,levels=kde.per)
    kc$id<-uniqid

    #writeOGR(kc,  dsn="utilization_distributions", layer = paste0(uniqid,"_kde"), driver =
"ESRI Shapefile")
    #for KDEline output, uncomment above
    print(u)
    capture.output(h.val,file="utilization_distributions/ud_output.txt",append=T)
    }


    #begin hole removal - in case the home range has holes (not the case in this paper) so
that area doesn't get counted, and to turn it into #a polygon
    ct<-0
    vertsort<-sort(kde.per,decreasing=T)
    #loop through each KDE level
    for (lev in vertsort) {
     ct<-ct+1
     t<-subset(kc,kc@data$level==lev)

     #convert to poly
     t1<-
SpatialPolygonsDataFrame(gPolygonize(t),data=data.frame(level=seq(1,length(gPolygonize
(t)),1)))
     t2<-unionSpatialPolygons(t1,IDs=rep(as.character(ct),length(t1)))

     holetab<-data.frame(id=NA,index=NA)

     for (i in t1@data$level)
     {
      l<-length(t1@polygons[[i]]@Polygons)
      if (l == 1) {next} else {
       for (p in 1:length(t1@polygons[[i]]@Polygons)) {
        polys<-t1@polygons[[i]]@Polygons
        poly<-t1@polygons[[i]]@Polygons[[p]]
        if (isTRUE(poly@hole)) {
         holetab<-rbind(holetab,c(i,p))
        }
       }
      }
```

```
    }

    holetab<-holetab[complete.cases(holetab),]
    if (length(holetab[,1]) == 0) {
      out<-SpatialPolygonsDataFrame(t2,data=data.frame(row.names=ct,level=lev))
      assign(paste0('kernel',lev),out)
      next
    }

    for (m in 1:length(holetab[,1]))
    {
      ind<-holetab[m,]
      hole<-t1@polygons[[ind$id]]@Polygons[[ind$index]]
      hole@hole<-FALSE
      hole<-Polygons(list(hole),'1')
      hole2<-SpatialPolygons(list(hole))
      t2<-gDifference(t2,hole2,id=as.character(ct))
    }

    out<-SpatialPolygonsDataFrame(t2,data=data.frame(row.names=ct,level=lev))
    assign(paste0('kernel',lev),out)
  }

  #browser()
  if (length(vertsort) > 1) {
    str<-paste('kde.out<-rbind(',paste(paste('kernel',vertsort,sep=""),collapse=','),')',sep="")
  } else {
    str<-paste('kde.out<-',paste('kernel',vertsort,sep=""),sep="")
  }

  eval(parse(text=str))
  #end hole removal

  #writePolyShape(kde.out, paste0("utilization_distributions/",uniqid,"_kde")) #maptools
method
  kde.out@data <- data.frame(id = uniqid, level = kde.out$level, type = "KDE")
  writeOGR(kde.out,  dsn="utilization_distributions", layer = paste0(uniqid,"_kde"), driver
= "ESRI Shapefile")


 } else {

capture.output(c(uniqid,length(alllocs$x),length(locs$x)),file="utilization_distributions/ud_
output.txt",append=T)
```

```
      print("Less than 20 mean daily locations, no kernel UD calcualated.")}

   } else {

capture.output(c(uniqid,length(alllocs$x),length(locs$x)),file="utilization_distributions/ud_
output.txt",append=T)
     print("Less than 5 total locations, no MCP calculated.")}
  }

}
writeOGR(mdl.shp, dsn="utilization_distributions", layer = "mean_daily_locs_all", driver =
"ESRI Shapefile")
save.image("utilization_distributions/ud_rWorkspace.Rdata")


#### ELEVATION AT KDEs ######


library(elevatr)
library(sf)

#### ----- load data as a Spatial Polygons Data Frame
kdeID <- readOGR(dsn = "utilization_distributions", layer = "ID_breeding_kde")
crs(kdeID) <- sf::st_crs('ESRI:102008')$proj4string  #set coordinate system
#get elevation data, elevation is in meters
kdeID_elev_aws <- get_elev_raster(locations = kdeID, src = "aws", z = 10)
#extract the standard deviation value for each 50% and 95% KDE polygon
kdeID_ex_95 <- raster::extract(kdeID_elev_aws, kdeID[kdeID$level == 95,], fun = sd) #put
KDE level in as needed


#### UNIVARIATE LINEAR REGRESSIONS ####

library(lme4)
library(performance)


#single variable models...
Lm_sex <- lm(Area_ha_50KDE ~ Sex,
          data = kdedata)

Lm_elev_cen <- lm(Area_ha_50KDE ~ elevation,
              data = kdedata)
```

```
Lm_elev_sd <- lm(Area_ha_50KDE ~ kdes50_sd,
             data = kdedata)

Lm_lat <- lm(Area_ha_50KDE ~ POINT_Y,
           data = kdedata)

Lm_shrub <- lm(Area_ha_50KDE ~ percShrub50,
            data = kdedata)

Lm_age <- lm(Area_ha_50KDE ~ Age,
           data = kdedata)


plot(Lm_elev_sd_50) #repeated for each one
model_performance(Lm_elev_sd, metrics = "all", verbose = TRUE) #repeated for each one



#### DEGREE OF OVERLAP IN HOME RANGES ####

#load libraries
library(lme4)
library(dplyr)
library(amt)

#here, all_data is a dataframe, and the IDs for which overlap is being assessed are 49189
and 49776

tracks <- amt::make_track(all_data, POINT_X, POINT_Y, DATE, id = BirdID)

trast <- amt::make_trast(tracks |> filter(id %in% c(49189, 49776)), res = 50)

hr_49189 <- hr_kde(tracks[tracks$id == 49189,], trast = trast, levels = c(0.5, 0.95))
plot(hr_49189)
hr_49776 <- hr_kde(tracks[tracks$id == 49776,], trast = trast, levels = c(0.5, 0.95))
plot(hr_49776)

hr_overlap(hr_49189, hr_49776, type = "ba", conditional = TRUE)
hr_overlap(hr_49189, hr_49776, type = "ba")
```
